# RNase Z Oxidative Degradation Impedes tRNA Maturation and is Involved in Streptococcal Translation Regulation in Response to Oxidative Stress

**DOI:** 10.1128/Spectrum.01167-21

**Published:** 2021-10-27

**Authors:** Yuzhu Dong, Huichun Tong, Qingqing Hu, Xiuzhu Dong

**Affiliations:** a State Key Laboratory of Microbial Resources, Institute of Microbiology, Chinese Academy of Sciences, Beijing, China; b University of Chinese Academy of Sciences, Beijing, China; Northwestern University

**Keywords:** RNase Z, *Streptococcus*, hydrogen peroxide stress, oxidative degradation, tRNA 3′ end maturation, translation repression

## Abstract

When encountering oxidative stress, organisms selectively upregulate antioxidant genes and simultaneously suppress the translation of most other proteins. Eukaryotes employ multiple strategies to adjust translation at both the initiation and elongation stages; however, how prokaryotes modulate translation under oxidative stress remains unclear. Here, we report that upon hydrogen peroxide (H_2_O_2_) challenge, Streptococcus oligofermentans reduced translation via RNase Z (So-RNaseZ) oxidative degradation, thus hindering tRNA maturation. S. oligofermentans encodes all CCA-less tRNAs that require So-RNaseZ for 3′ end maturation. A combination of nonreducing SDS-PAGE and liquid chromatography/tandem mass spectrometry (LC/MS-MS) assays demonstrated that H_2_O_2_ oxidation induced Cys38-Cys149 disulfide linkages in recombinant So-RNaseZ protein, and serine substitution of Cys38 or Cys149 abolished these disulfide linkages. Consistently, redox Western blotting also determined intramolecular disulfide-linked So-RNaseZ in H_2_O_2_-treated *S. oligofermentans* cells. The disulfide-linked So-RNaseZ and monomer were both subject to proteolysis, whereas C149S mutation alleviated oxidative degradation of So-RNaseZ, suggesting that H_2_O_2_-mediated disulfide linkages substantially contributed to So-RNaseZ degradation. Accordingly, Northern blotting determined that tRNA precursor accumulation and mature tRNA species decrease in H_2_O_2_-treated *S. oligofermentans*. Moreover, reduced overall protein synthesis, as indicated by puromycin incorporation, and retarded growth of *S. oligofermentans* occurred in an H_2_O_2_ concentration-dependent manner. Overexpression of So-RNaseZ not only elevated tRNA precursor processing and protein synthesis but also partly rescued H_2_O_2_-suppressed *S. oligofermentans* growth. Moreover, So-RNaseZ oxidative degradation-mediated translation repression elevated *S. oligofermentans* survival under high H_2_O_2_ stress. Therefore, this work found that So-RNaseZ oxidative degradation-impeded tRNA maturation contributes to streptococcal translation repression and provides the oxidative stress adaptability for *S. oligofermentans*.

**IMPORTANCE** Translation regulation is a common strategy used by organisms to reduce oxidative damage. Catalase-negative streptococci produce as well as tolerate high levels of H_2_O_2_. This work reports a novel translation regulation mechanism employed by Streptococcus oligofermentans in response to H_2_O_2_ challenge, in which the key tRNA endonuclease So-RNaseZ is oxidized to form Cys38-Cys149 disulfide linkages and both the disulfide-linked So-RNaseZ and monomers are subject to proteolysis; thus, tRNA maturation, protein translation, and growth are all suppressed. Notably, So-RNaseZ oxidative degradation-mediated translation repression offers oxidative adaptability to *S. oligofermentans* and enhances its survival against high H_2_O_2_ challenge. So-RNaseZ orthologs and H_2_O_2_-sensitive cysteines (Cys38 and Cys149) are widely distributed in *Streptococcus* and *Lactococcus* species genomes, which also encode all CCA-less tRNAs and lack catalase. Therefore, RNase Z oxidative degradation-based translation regulation could be widely employed by these lactic acid bacteria, including pathogenic streptococci, to cope with H_2_O_2_.

## INTRODUCTION

Reactive oxygen species (ROS), including hydrogen peroxide (H_2_O_2_), superoxide anions (O_2_^−^), and hydroxyl radicals (•OH), are universal environmental stressors for almost all types of organisms (1, 2). Therefore, prokaryotes have developed a cohort of strategies to protect themselves from ROS damage, including the synthesis of ROS detoxifying enzymes like superoxide dismutase (SOD) and catalase to decompose O_2_^−^ and H_2_O_2_ and controlling cellular metal homeostasis to avoid Fenton reaction-triggered formation of more harmful •OH (3, 4). Expression of antioxidant genes is usually regulated by specific ROS-responding transcriptional regulators (3). Detailed molecular mechanisms of antioxidative stress have been elucidated in model species, such as Escherichia coli that employs the *oxyR* and *soxRS* regulons to defend against damage from H_2_O_2_ and O_2_^−^, respectively (5, 6), while Bacillus subtilis utilizes PerR as a global oxidant sensor and transcriptional regulator (7). In addition to upregulating the expression of antioxidant functional genes, organisms must swiftly modulate their basic biological processes to reduce oxidative damage to proteins and DNA, like transiently and reversibly inhibiting translation when cells encounter oxidative stress (8).

Translation regulation is widely used by organisms to cope with various environmental stresses, such as hypoxia, nutritional starvation, and oxidative stress (8–12). When exposed to oxidative stress, eukaryotes regulate translation either at initiation stage through phosphorylating initiation factor 2 α subunit (eIF2α) to inhibit translational initiation complex assembly (8, 13) or at elongation stage by stalling ribosomes at tryptophan codons and promoting the phosphorylation of elongation factor eEF2 or cleaving tRNAs to slow down translation rates (14–18). Knowledge on how prokaryotes regulate translation in response to oxidative stress is scant. The limited data have indicated that E. coli inhibits translation elongation and protein synthesis by rapidly degrading tRNAs when exposed to H_2_O_2_ (19, 20).

Streptococci are catalase-negative facultative anaerobic bacteria and are well known for producing and tolerating high levels of H_2_O_2_ (21–24). Similar to E. coli and B. subtilis, streptococci are capable of regulating antioxidant genes in response to H_2_O_2_ (25–28). For example, Streptococcus pneumoniae, a human opportunistic pathogen, employs the response regulator RR14 to sense H_2_O_2_ and derepress the expression of antioxidant genes (27), while Streptococcus oligofermentans, an oral commensal bacterium, utilizes the peroxide-responsive regulator PerR cysteine oxidative inactivation to derepress the expression of oxidative stress defense genes (28, 29). Previously, we found that *S. oligofermentans* would pause growth for a period of time when encountering H_2_O_2_ (28), implying that translation regulatory mechanisms could be involved in adaptation to oxidative stress. Reprogramming translation could be more significant for catalase-negative streptococci because of their inability to decompose cellular H_2_O_2_ efficiently (22, 28). In our previous unpublished quantitative proteomic study, we found significantly decreased levels of RNase Z, which specifically processes the 3′ end of tRNA precursors for maturation in 0.5 mM H_2_O_2_-treated *S. oligofermentans*, and this could impede translation efficiency as a result of reduced mature tRNA pools.

In this study, we determined that in H_2_O_2_-treated *S. oligofermentans*, RNase Z from S. oligofermentans (So-RNaseZ) was oxidized to form intramolecular Cys38-Cys149 disulfide linkages and that both the disulfide-linked So-RNaseZ and monomers are subject to proteolysis. Decreased levels of the cellular So-RNaseZ caused accumulation of tRNA precursors and reduction of mature tRNA pools. Accordingly, overall protein synthesis and growth were suppressed in an H_2_O_2_-dose-dependent manner. Overexpression of So-RNaseZ elevated tRNA precursor processing and translation efficiency and partly rescued growth of H_2_O_2_-challenged *S. oligofermentans*. Moreover, So-RNaseZ oxidative degradation-mediated translation repression elevated the survivability of *S. oligofermentans* in higher H_2_O_2_. The So-RNaseZ orthologs, and Cys38 and Cys149, are widely distributed in *Streptococcus* and *Lactococcus* spp.; therefore, this RNase Z oxidation-mediated translation regulatory strategy could be widely used in catalase-negative bacteria.

## RESULTS

### *S. oligofermentans* tRNAs can be processed exclusively by So-RNaseZ for 3′ end maturation.

**FIG 1:**
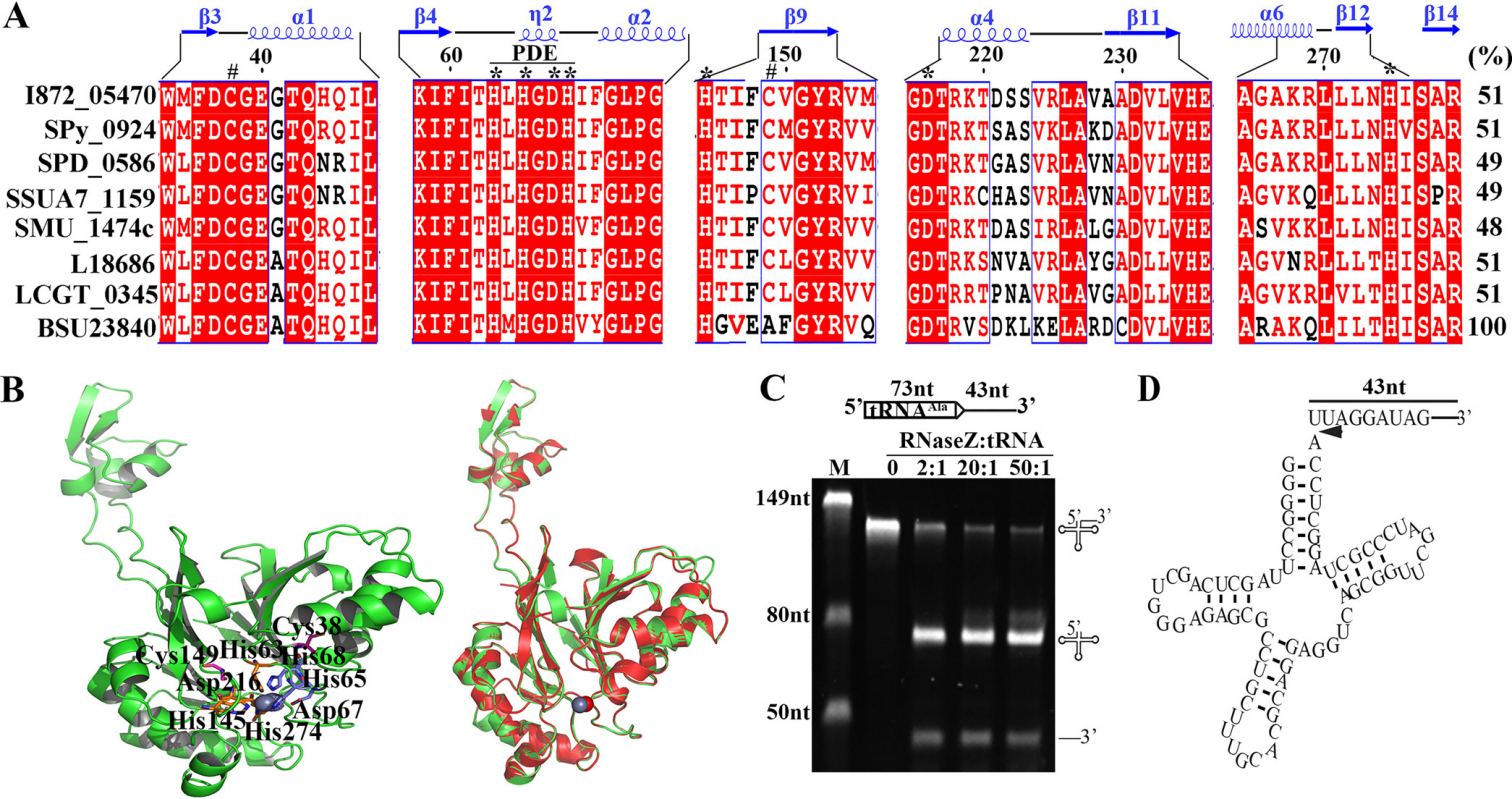
The *S. oligofermentans* So-RNaseZ processes the 3′ ends of tRNA precursors for mutation. (A) Sequence alignment of So-RNaseZ and the orthologs from Streptococcus, *Lactococcus*, and B. subtilis. Protein sequences were retrieved from the KEGG database and aligned using ClustalW. I872_05470: *S. oligofermentans*; Spy_0924: S. pyogenes; SPD_0586: S. pneumoniae; SSUA7_1159: S. suis; SMU_1474c: S. mutans; L18686: Lactococcus lactis; LCGT_0345: L. garvieae; and BSU23840: B. subtilis. The PDE domain, conserved amino acid residues binding Zn^2+^ (*), and cysteine residues at positions 38 and 149 (#) are indicated. The secondary structure of B. subtilis RNase Z and the So-RNaseZ amino acid residue numbers are shown at the top. The rightmost numbers represent the amino acid identities (%) of respective protein with BSU23840. (B) Homology modeling of the So-RNaseZ structure was constructed using SWISSMODEL by automatically selecting the B. subtilis RNase Z (4GCW) as a template. Conserved cysteine residues and amino acid residues for Zn^2+^ (sphere) binding are shown in the left panel, and superimposition of the So-RNaseZ homology model over 4GCW is shown in the right panel. (C) The 116 nt tRNA^Ala^ (I872_t10692) precursor RNA (schematic at top) was produced via *in vitro* transcription, and 50 ng of tRNA precursor was mixed with a gradient of increasing So-RNaseZ protein at indicated molar ratios. Detailed procedures are described in Materials and Methods. The endonucleolytic products were examined on 10% urea-PAGE gels. M, RNA marker with molecular weights indicated on the gel left; the precursor and cleaved product symbols are shown on the gel right. (D) Schematic of the tRNA^Ala^ precursor showing 5′ and 3′ RACE-determined So-RNaseZ cleavage sites located immediately downstream of the discriminator nucleotide and indicated by an arrow.

RNase Z, a member of the metallo-beta-lactamase superfamily, removes the 3′ trailers of tRNA precursors by cleaving immediately downstream of the discriminator nucleotide and implements 3′ end maturation of tRNAs lacking encoded CCA motif (30–34). Previously, we found that the *S. oligofermentans* RNase Z (KEGG accession no.: I872_05470) abundance significantly decreased in 0.5 mM H_2_O_2_-treated cells. I872_05470 displays 51% amino acid identity with the Bacillus subtilis RNase Z (BSU23840) (Fig. 1A). Similar to BSU23840 (30), I872_05470 possesses the conserved phosphodiesterases (PDE) domain and Zn^2+^-binding residues, His63, His65, Asp67, His68, His145, Asp216, and H274 (Fig. 1A and B). Moreover, homology modeling showed a perfect structural concordance between So-RNaseZ and BSU23840, especially at the long flexible protruding arm that is believed to bind to the TψC arm of tRNAs (35) (Fig. 1B). Thus, I872_05470 was tentatively named So-*rnaseZ* and So-RNaseZ was used for the encoded protein.

*S. oligofermentans* possesses 50 tRNA genes in total, and all are CCA-less (Fig. S1A); thus, they should be processed exclusively by So-RNaseZ for 3′ end maturation. Accordingly, the genomic So-*rnaseZ* could be deleted only when So-*rnaseZ* was ectopically expressed, implying the indispensability of So-RNaseZ for *S. oligofermentans*. To confirm that So-RNaseZ processes the 3′ trailers of tRNA precursors, we used five pre-tRNAs as the substrates, which are cotranscribed with other genes and thus would produce longer 3′ extensions for assay convenience. Figure 1C shows that with the addition of increasing concentrations of recombinant So-RNaseZ protein, a 116-nucleotide (nt) tRNA^Ala^ transcript, composed of a 73-nt tRNA^Ala^ (I872_t10692) and a 43-nt 3′ trailer, was cleaved into two fragments with lengths of 73 nt and 43 nt, respectively, and elevated product yields occurred as the protein contents increased. Furthermore, 5′ rapid amplification of cDNA ends (RACE) identified a uridine nucleotide (U) at the 5′ end of the 43-nt fragment, whereas 3′ RACE demonstrated an adenine nucleotide (A) at the 73-nt fragment 3′ end, verifying that So-RNaseZ cleaves the tRNA^Ala^ precursor immediately downstream of the discriminator nucleotide (Fig. 1D). Similarly, So-RNaseZ processed the 3′ ends of tRNA^Pro^ (I872_t10754), tRNA^Met^ (I872_t10730), tRNA^Leu^ (I872_t10780), and tRNA^Arg^ (I872_t10790) precursors in exactly the same pattern as that used with the tRNA^Ala^ precursor (Fig. S2). Therefore, these nucleolytic assays confirmed that So-RNaseZ functions in processing the 3′ trailers of tRNA precursors for 3′ end maturation.

### H_2_O_2_ treatment causes Cys38-Cys149 disulfide linkages in So-RNaseZ.

**FIG 2:**
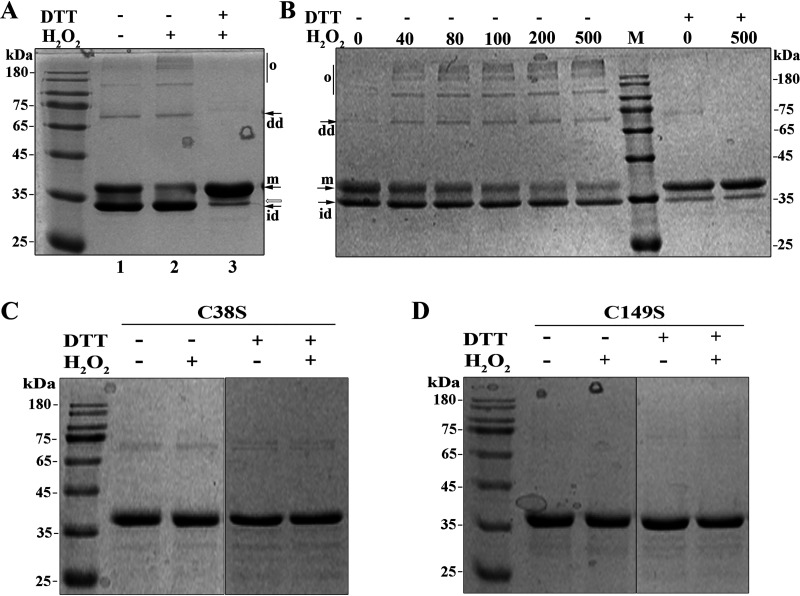
Nonreducing SDS-PAGE examines H_2_O_2_ oxidation-caused Cys38-Cys149 disulfide linkages in So-RNaseZ. (A) The recombinant So-RNaseZ was treated with or without 500 μM H_2_O_2_ for 30 min and subsequently reduced with 10 mM DTT. (B) The recombinant So-RNaseZ was treated with a gradient of concentrations of H_2_O_2_ (μM) for 30 min and subsequently reduced with 10 mM DTT. M, molecular weight marker; m, So-RNaseZ monomer; id, intramolecular disulfide linkage; dd, disulfide-linked dimer; and o, disulfide-linked oligomers. Empty arrow indicates the band containing a presumed different conformation of So-RNaseZ monomer as identified by MALDI-TOF (Fig. S3). (C and D) Cys38 and Cys149 of So-RNaseZ were each mutated into serine and mutant proteins were overexpressed in E. coli. Purified So-RNaseZC38S (C38S) (C) and So-RNaseZC149S (C149S) (D) were treated with or without 500 μM H_2_O_2_ for 30 min and subsequently reduced using 10 mM DTT. The statuses of wild-type and cysteine-mutated So-RNaseZ proteins were examined by 12% nonreducing SDS-PAGE. Molecular weight marker is shown beside the gel. Experiments were repeated at least three times, and representative results are shown.

Given that significantly decreased So-RNaseZ was found in H_2_O_2_-treated *S. oligofermentans* in our unpublished quantitative proteomic study, and H_2_O_2_-mediated cysteine oxidation led to the degradation of the *S. oligofermentans* MntR protein (36), we tested whether the cysteine residues of So-RNaseZ were oxidized by H_2_O_2_. Recombinant So-RNaseZ was purified in the presence of 1 mM ethylenediaminetetraacetic acid (EDTA) and treated with 5 g/liter Chelex 100 to remove metal ions. The purified So-RNaseZ was treated with H_2_O_2_, and then the oxidation status of So-RNaseZ was examined on 12% nonreducing SDS-PAGE gels. Compared with non-H_2_O_2_-treated So-RNaseZ (Fig. 2A, lane 1), a 30-min treatment with 500 μM H_2_O_2_ caused a significant decrease in the content of the So-RNaseZ monomer, but the bands of presumable oligomers and dimers and assumed intramolecular disulfide-linked monomer, which migrated faster than the So-RNaseZ monomer, appeared (Fig. 2A, lane 2); dithiothreitol (DTT) treatment, however, abolished all of the assumed oligomers, dimers, and the intramolecular disulfide-linked band (Fig. 2A, lane 3), indicating that H_2_O_2_ oxidizes So-RNaseZ to form intra- and intermolecular disulfide linkages. However, a slightly faster migrating band was retained even after DTT reduction (Fig. 2A, lane 3, empty arrow indicated), which was identified as So-RNaseZ by matrix-assisted laser desorption ionization–time of flight mass spectrometry (MALDI-TOF) (Fig. S3A). Thus, a different So-RNaseZ protein conformation, mediated by H_2_O_2_ treatment, was presumed. Notably, disulfide-linked mono-, di-, and oligomers were already present before H_2_O_2_ treatment even if the protein was purified in the presence of 1 mM DTT (lane 1), suggesting that So-RNaseZ could be highly sensitive to air oxidization. This is verified to be the case, because as low as 40 μM H_2_O_2_ treatment induced oligomer and dimer formation of So-RNaseZ (Fig. 2B).

To further verify the presence of disulfide linkages in H_2_O_2_-treated So-RNaseZ, the oligomer, dimer, and intramolecular disulfide-linked bands in Figure 2A, lane 2, were excised and trypsin digested. LC/MS-MS analysis identified Cys38-Cys149- and Cys149-Cys149-linked peptides in the predicted disulfide-linked monomer and dimer and oligomer bands, respectively. The representative MS/MS spectrometric maps (Fig. S3B) showed the disulfide linkage details, such as the intramolecular Cys38-Cys149 was identified as a 6-charged peptide fragment of LLEEINEVWMFDC_38_GEGTQHQILETTIKPR-LDHTIFC_149_VGYR (precursor mass of 4,766.3212), while intermolecular Cys149-Cys149 was identified as a 4-charged peptide fragment (FTVYADKLDHTIFC_149_VGYR-LDHTIFC_149_VGYR) (precursor mass of 3,468.7258).

Next, we substituted So-RNaseZ Cys38 and Cys149 with serine and then examined them for H_2_O_2_-induced disulfide linkages. Nonreducing SDS-PAGE assays showed that C38S mutation almost, and C149S mutation completely, abolished disulfide-linked monomer, dimer, and oligomer bands (Fig. 2C and D). Of note, an ∼70 kDa protein band, which could be reduced by DTT, occurred in C38S-mutated protein and was predicted to be a Cys149-Cys149 disulfide-linked dimer. These results demonstrated that Cys38 and Cys149 are the H_2_O_2_ oxidization targets in the formation of intra- and intermolecular disulfide linkages.

### H_2_O_2_-induced disulfide linkages result in the degradation of cellular So-RNaseZ.

**FIG 3:**
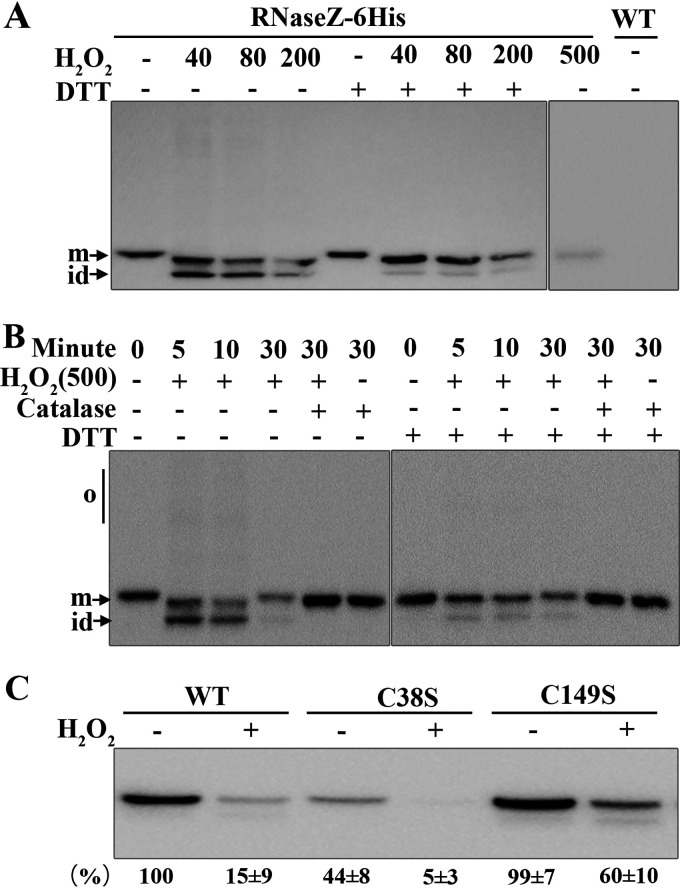
Examination of disulfide linkages and degradation of So-RNaseZ in H_2_O_2_-treated *S. oligofermentans*. (A and B) Mid-exponential-phase anaerobically grown RNaseZ-6×His cells, which carry a So-RNaseZ C-terminal fusion with a 6×His tag, were 30 min treated without (−) or with 40, 80, 200, and 500 μM H_2_O_2_ (A), or treated with 500 μM H_2_O_2_ for different time periods and concomitantly with 1 KU catalase or not (B). Cells were then lysed, and one aliquot was reduced with 10 mM DTT. All samples were run on 10% nonreducing SDS-PAGE gels, and So-RNaseZ was examined by Western blotting using anti-His antibody. The wild-type strain (WT) was included as a negative control. o, oligomers; m, monomer; and id, intramolecular disulfide-linked monomer. All experiments were repeated three times, and representative images are shown. (C) Wild-type So-RNaseZ6×His (WT) and C38S- and C149S-mutated So-RNaseZ6×His complemented strains were treated with 500 μM H_2_O_2_ for 30 min, and the cellular levels of So-RNaseZ were examined by Western blotting using anti-His antibody. Band intensities of the So-RNaseZ protein were measured using Image J and expressed as percentages of WT cell lysate without treatment with H_2_O_2_. The experiments were repeated three times, and the averages ± standard deviation (SD) are shown below a representative image.

To examine the status of So-RNaseZ in H_2_O_2_-treated *S. oligofermentans* cells, we constructed an RNaseZ-6×His strain, which carried a 6×His tag at the C terminus of So-RNaseZ. The mid-exponential-phase cells of anaerobically grown RNaseZ-6×His were then divided into five aliquots. Four aliquots were 30 min treated with 40, 80, 200, and 500 μM H_2_O_2_, respectively, leaving the other aliquot untreated as a control. Cells were then sonicated in the presence of *N*-ethylmaleimide (NEM) to chelate protein thiol groups and in the presence of EDTA to chelate Fe^2+^, thus avoiding Fenton chemistry-mediated oxidization. The oxidation status of So-RNaseZ in each treatment was then examined by redox Western blotting. As shown in Figure 3A, a protein band migrating faster than the So-RNaseZ monomer occurred in 40 μM, 80 μM, and 200 μM H_2_O_2_-treated cells, and this band could be reduced by DTT. This result indicated that cellular So-RNaseZ was oxidized to form intramolecular-linked disulfide in H_2_O_2_-treated bacterium. It is worth noting that the So-RNaseZ content of either the intramolecular disulfide linkage or the monomer decreased in 200 μM H_2_O_2_-treated cells, whereas the former completely disappeared in 500 μM H_2_O_2_-treated cells (Fig. 3A), suggesting that H_2_O_2_-oxidized So-RNaseZ could be subjected to proteolysis. Furthermore, intramolecular disulfide linkages occurred after 5 min treatment of 500 μM H_2_O_2_ and gradually decreased until they disappeared 30 min posttreatment (Fig. 3B). The intramolecular disulfide linkages were eliminated by a concomitant addition of catalase to the culture or by the addition of DTT to H_2_O_2_-treated cell lysates (Fig. 3B). Similarly, the slightly faster migrating protein band was maintained even after DTT reduction as observed in H_2_O_2_-treated recombinant So-RNaseZ (Fig. 2A). So-RNaseZ was not detected in the cell pellets from all treatments, and So-*rnaseZ* transcript abundances did not change, as indicated by quantitative PCR (qPCR) assays (Fig. S4). Thus, these experimental data demonstrated that H_2_O_2_ treatment causes degradation of So-RNaseZ, most likely through disulfide linkage formation or cysteine oxidation.

To further verify that it was disulfide-linked So-RNaseZ that was subjected to proteolysis, the recombinant So-RNaseZ protein was first treated with H_2_O_2_ or not and then 30 min coincubated with the cell lysate of *S. oligofermentans* at 37°C. Western blotting determined a significant decrease of the H_2_O_2_-treated So-RNaseZ protein compared with the untreated one (Fig. S5). Therefore, the disulfide-linked aberrant So-RNaseZ would be rapidly removed by proteases.

Next, H_2_O_2_ oxidization-caused So-RNaseZ degradation was further verified in cysteine-mutated protein. Wild-type and C38S- and C149S-mutated So-*rnaseZ*-6×His genes were integrated into the shuttle plasmid pDL278 (37) and transformed into the wild-type strain. Subsequently, the genomic So-*rnaseZ* was deleted in these strains and then the cellular abundance of So-RNaseZ was examined by Western blotting. Upon H_2_O_2_ treatment, only 15% of wild-type So-RNaseZ was retained, whereas 60% of So-RNaseZC149S was detected (Fig. 3C), indicating that Cys149 could be an oxidation target and contributes to H_2_O_2_-oxidized So-RNaseZ degradation. Markedly reduced So-RNaseZC38S, however, was found in H_2_O_2_-untreated cells (Fig. 3C), likely because Cys38 substitution changed the protein conformation, thus causing degradation. The conformation change of So-RNaseZC38S was confirmed by circular dichroism spectroscopy analysis (Fig. S6). Nevertheless, this makes assaying the H_2_O_2_ sensitivity of Cys38 through residue substitution impossible. Furthermore, even if anaerobically growing the RNaseZ-6×His cells in chemically defined medium lacking Fe^2+^, and thus preventing Fenton chemistry-induced protein damage, H_2_O_2_ treatment still caused significant degradation of So-RNaseZ (Fig. S7). This result indicated that H_2_O_2_, but not Fenton chemistry-generated hydroxyl radicals, causes the cysteine oxidization and degradation of So-RNaseZ. Of note, 40% protein degradation was still observed in H_2_O_2_-treated So-RNaseZC149S, indicating that although disulfide linkages cause substantial degradation of So-RNaseZ, other proteolytic mechanisms also might be involved.

### H_2_O_2_ treatment impedes tRNA maturation of *S. oligofermentans*.

**FIG 4:**
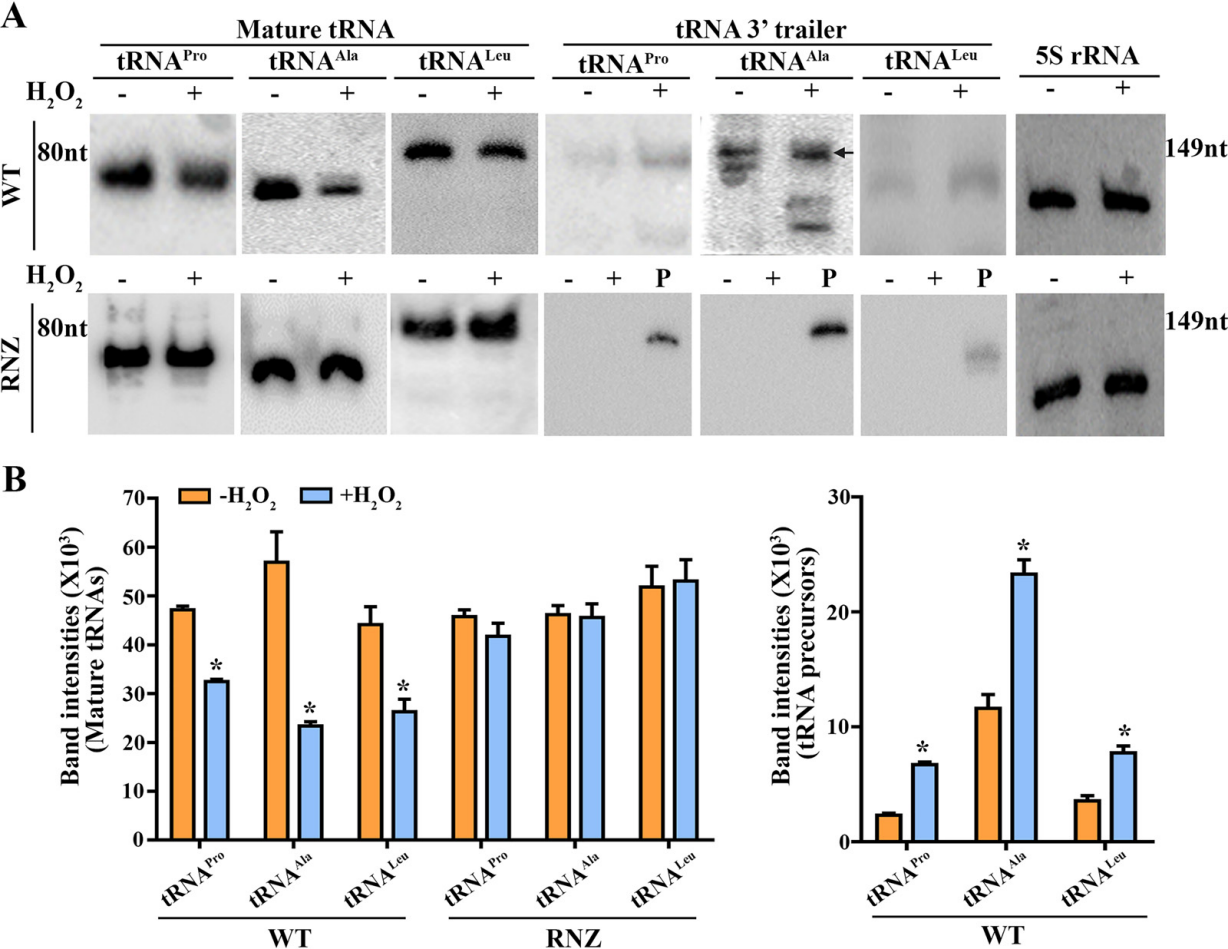
Northern blotting assays H_2_O_2_ treatment-caused tRNAs abundance changes in *S. oligofermentans* and overexpression of So-RNaseZ on tRNAs processing. (A) The anaerobically grown mid-exponential-phase wild-type (WT, upper panel) and So-RNaseZ-overexpressing (RNZ; lower panel) cells were treated with or without 500 μM H_2_O_2_ for 30 min and then collected for total RNA extraction. Northern blotting was used to examine the mature and precursor levels of three representative tRNAs using probes targeting the corresponding mature and 3′ trailer tRNA sequences as indicated at the gel tops, respectively. Total RNA of 0.5 μg and 10 μg was loaded for detecting mature tRNAs and precursors, respectively. 5S rRNA hybridization was used as a loading control. P, 40 ng of *in vitro* transcribed respective tRNA precursor fragment, composed of mature and 3′ trailer tRNA sequence, was loaded as a positive control. Black arrow indicates the position of the tRNA^Ala^ precursor, and RNA molecular size markers are shown on the side. Triplicate experiments were performed and representative images are shown. (B) Northern blotting signal intensities of RNA bands in H_2_O_2_-treated and -untreated samples in panel A were measured using Image J. The bar diagrams show the levels of mature tRNA (left) and tRNA precursors (right) in H_2_O_2_-treated and -untreated samples. No tRNA precursors were detected in So-RNaseZ-overexpressing cells treated with or without H_2_O_2_. The averages ± SD from triplicate experiments are shown. *, significantly different from the level of the corresponding tRNA species in anaerobically grown cells (Student’s *t* test; *P < *0.05).

Given that all *S. oligofermentans* tRNAs are CCA-less and presumably processed by So-RNaseZ, accumulation of tRNA precursors but decrease of mature tRNAs were predicted in H_2_O_2_-treated *S. oligofermentans* because of So-RNaseZ degradation. To verify this assumption, three representative tRNAs [tRNA^Pro(TGG)^, I872_t10754; tRNA^Ala(TGC)^, I872_t10692; and tRNA^Leu(CAG)^, I872_t10780] were selected for further examination. These three tRNAs not only have a terminator structure that prevents 3′–5′ exoribonuclease cleavage but also have long 3′ trailer sequences that allow precursor examination (Fig. S1B). As shown in Figure 4A, Northern blotting detected decreased mature tRNAs and accumulated precursors for the three tRNAs, in particular tRNA^Ala(TGC)^, in H_2_O_2_-treated *S. oligofermentans*. It is worth noting that total RNA 20-fold higher than that used for detecting mature species was used for detecting the precursors, most likely because the nonmatured tRNA precursors were more liable to be degraded by ribonucleases (38). Supportively, upon incubation of *in vitro* transcribed tRNA precursors with cell lysates from anaerobically cultured *S. oligofermentans* treated with or without H_2_O_2_, a rapid degradation of all three tRNA precursors was observed (Fig. S8). Comparing the ratios of precursor or mature contents of a given tRNA in H_2_O_2_-treated versus -untreated cells, we found an approximately 2.0- to 2.8-fold higher precursor content but a 1.45- to 2.41-fold lower content of mature species for all three tested tRNAs (Fig. 4B). This indicates that H_2_O_2_ oxidation hinders the tRNA maturation of *S. oligofermentans*.

### *S. oligofermentans* suffers translation and growth repression under high concentrations of H_2_O_2_.

**FIG 5:**
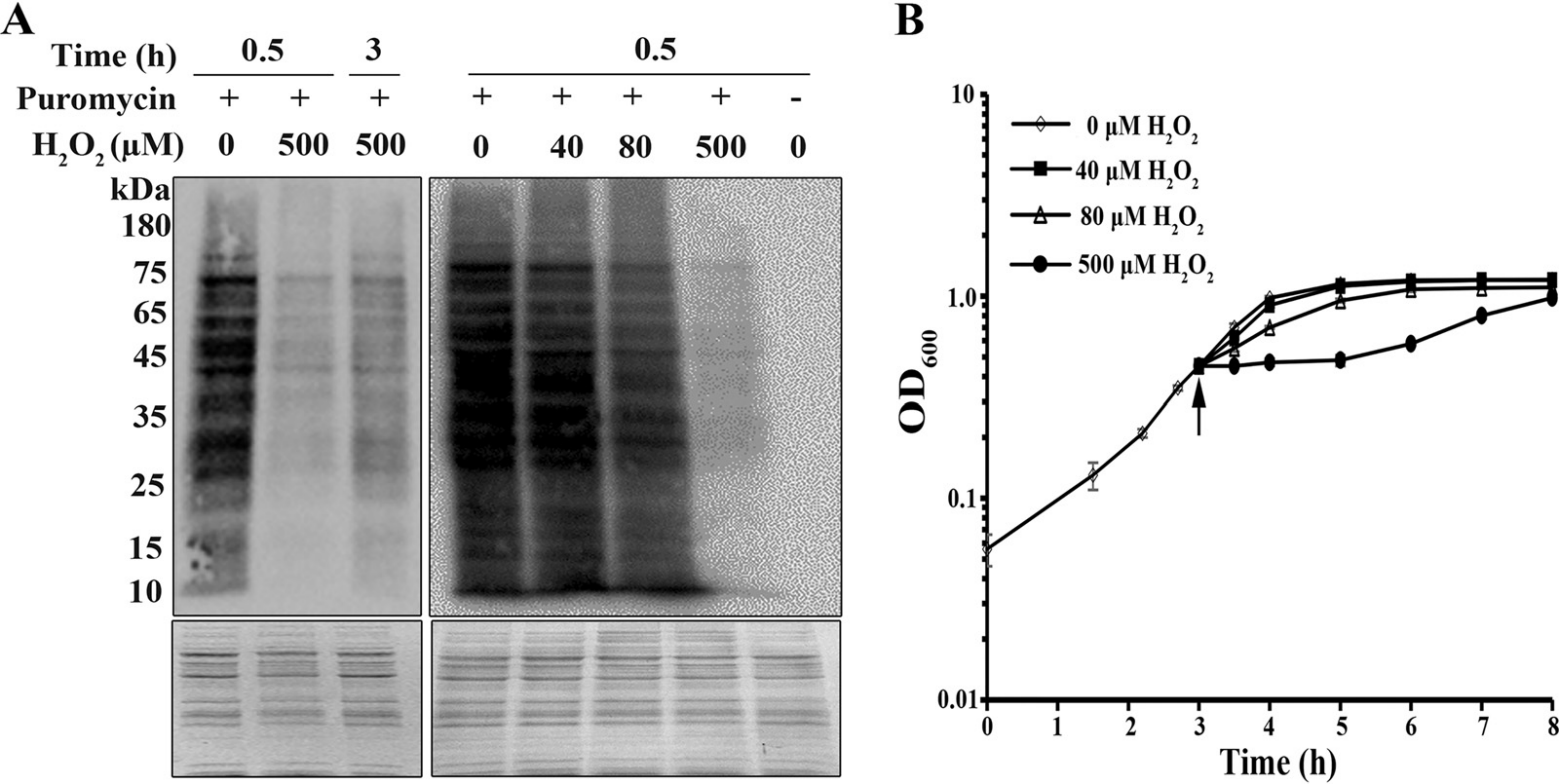
Determination of the overall protein synthesis via puromycin integration and growth of H_2_O_2_-treated *S. oligofermentans*. (A) Mid-exponential-phase cells of anaerobically grown *S. oligofermentans* were treated with 0.5 mM H_2_O_2_ for indicated times (left panel) or with various concentrations of H_2_O_2_ for 30 min (right panel). Puromycin was added at a final concentration of 10 μg/ml 10 min before cell collection, and then cells were lysed. Same amounts of total cell protein were loaded on two 12% SDS-PAGE gels, and one was examined by Western blotting using anti-puromycin antibody (upper panel) and the other was stained with Coomassie brilliant blue as an indicator of equivalent loading (lower panel). Molecular weight marker is shown on the left. (B) *S. oligofermentans* was grown anaerobically until the OD_600_ reached 0.4, and cells were divided into four aliquots, with three added with 40, 80 and 500 μM H_2_O_2_, respectively, and the other left untreated. The OD_600_ was measured at indicated time points. Arrow indicates the time point of H_2_O_2_ addition. Triplicate experiments were performed, and either one representative result is shown (A) or the averages ± SD are shown (B).

Given that tRNA precursor accumulation and mature tRNA decrease occurred in 0.5 mM H_2_O_2_-treated *S. oligofermentans* cells, the effect of tRNA maturation defects on translation was examined using puromycin incorporation as an indicator. Puromycin, an aminonucleoside antibiotic and a tRNA analogue, covalently binds to a nascent polypeptide chain during active translation, so it can be used to estimate newly synthesized protein contents by using an anti-puromycin antibody (39, 40). First, the mid-exponential-phase cells of anaerobically grown *S. oligofermentans* were divided into two aliquots, with one aliquot treated with 0.5 mM H_2_O_2_ and the other remaining untreated. After 20 min incubation at 37°C, puromycin at a final concentration of 10 μg/ml was added to the two aliquots, and after another 10-min incubation, cells were collected for translation examination. Compared with those in H_2_O_2_-untreated cells, the overall puromycin-integrated proteins significantly decreased in both band numbers and abundances in 30 min H_2_O_2_-treated cells, while translation appeared to resume 3 h post-H_2_O_2_ treatment (Fig. 5A, left panel). This indicates that 0.5 mM H_2_O_2_ treatment inhibits overall protein translation. Next, the anaerobic cultures were treated with different concentrations of H_2_O_2_, and Western blotting assayed decreased, although only slightly, overall intensities of puromycin hybridization signals in 40 μM H_2_O_2_-treated cells, but more evident reduction occurred in 80 μM H_2_O_2_-treated cells (Fig. 5A, right panel). This determined that even trace amounts of H_2_O_2_ inhibit translation.

Consistent with increased tRNA precursors and reduced translation in 0.5 mM H_2_O_2_-treated cells, growth of *S. oligofermentans* was also significantly inhibited (Fig. 5B), whereas no or only slight growth pausing was observed in 40 μM or 80 μM H_2_O_2_-challenged bacterium. Remarkably, growth resumed 3 h after 0.5 mM H_2_O_2_ treatment (Fig. 5B), in accordance with recovered protein synthesis over the same period (Fig. 5A). This result further indicates that H_2_O_2_-caused translational inhibition leads to growth pause.

### Overexpression of So-RNaseZ recovers tRNA 3′ end maturation and translation and partly rescues growth of H_2_O_2_-challenged *S. oligofermentans*.

**FIG 6:**
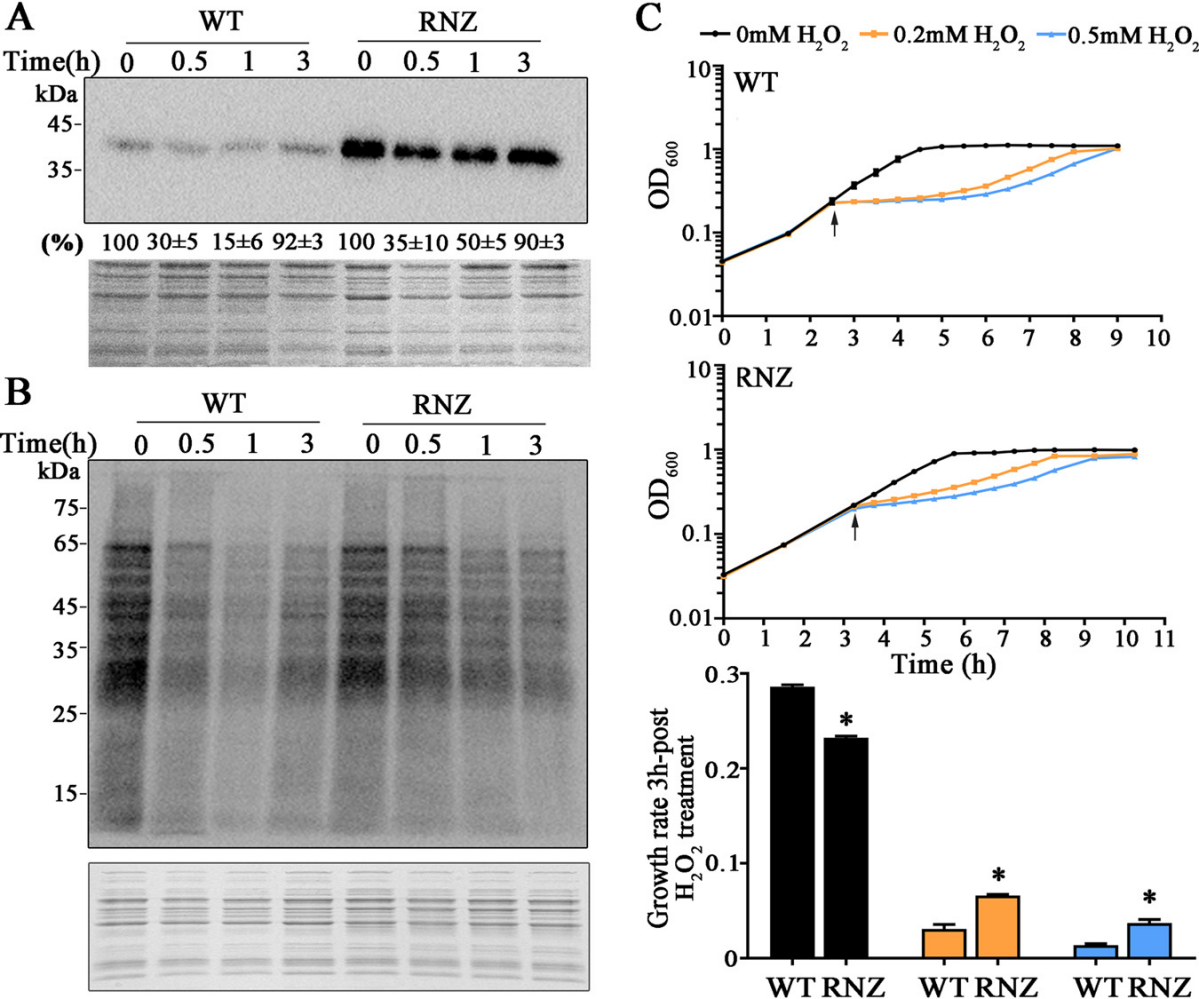
So-RNaseZ overexpression recovers H_2_O_2_-suppressed protein synthesis and growth of *S. oligofermentans*. (A) Western blotting assayed the So-RNaseZ abundances in strains pDL278RNaseZ-6×His (WT) and So-RNaseZover (RNZ). The two strains were cultured anaerobically, and until the mid-exponential phase, 500 μM H_2_O_2_ was added. After H_2_O_2_ treatment at indicated times, cells were collected and lysed. Same amounts of total cell protein were loaded on two 12% SDS-PAGE gels, and one was examined by Western blotting using anti-His antibody (upper panel), while the other was stained with Coomassie brilliant blue as an indicator of equivalent loading (lower panel). Western blotting signal intensities of the So-RNaseZ protein were measured using Image J and expressed as the percentages of respective strain without H_2_O_2_ treatment. Triplicate experiments were performed, and averages ± SD are shown below one representative image. (B) Strains were cultured and H_2_O_2_ treated as described in panel A, and puromycin was added 10 min before cell collection. Western blotting assayed puromycin integration using the anti-puromycin antibody (upper panel), and the same amounts of the total protein were run on 12% SDS-PAGE gels that were stained with Coomassie brilliant blue (lower panel). Molecular weight marker is shown for each gel on the left. Triplicate experiments were performed, and representative results are shown. (C) Strains in panel A were grown anaerobically in triplicate to an OD_600_ of ∼0.2, two replicates were treated with 200 and 500 μM H_2_O_2_ (arrows pointed), respectively, and the other remained untreated. The growth of strains WT (top panel) and RNZ (middle panel) was monitored by measuring OD_600_ at indicated time points. The lowest panel shows the growth rates within 3 h post-H_2_O_2_ addition. Experiments were repeated three times on triplicate samples for each measurement, and averages ± SD of one independent experiment are shown. *, significantly different from the WT strain (Student’s *t* test, *P* <0.05).

To link translation and growth suppression in H_2_O_2_-treated *S. oligofermentans* with So-RNaseZ degradation-caused tRNA maturation impediments, the So*-rnaseZ*-6×His gene was fused to the constitutive l-lactate dehydrogenase (LDH) promoter, cloned into the shuttle plasmid pDL278, and transformed into the wild-type strain to construct the So-RNaseZ overexpressing strain (So-RNaseZover). A vacant pDL278 was transformed into strain RNaseZ-6×His to obtain pDL278RNaseZ-6×His as a nonoverexpression control. These two strains were anaerobically cultured and treated with or without 0.5 mM H_2_O_2_ for 30 min. As shown in Figure 6A, Western blotting determined that although similar So-RNaseZ degradation occurred in the two strains treated by 0.5 mM H_2_O_2_, So-RNaseZ abundance was about 10-fold higher in strain Soi-RNaseZover than in pDL278RNaseZ-6×His, confirming the overexpression of So-RNaseZ.

As expected, Northern blotting assayed neither tRNA precursor accumulation nor mature tRNA reduction in the 0.5 mM H_2_O_2_-treated So-RNaseZ overexpression strain (Fig. 4A, lower panel). Consistently, significantly higher puromycin integration signals were found in strain So-RNaseZover than in strain pDL278RNaseZ-6×His after 0.5 h treatment with 0.5 mM H_2_O_2_ (Fig. 6B), demonstrating that elevated levels of So-RNaseZ are capable of overcoming H_2_O_2_-caused translation suppression. It is worth noting that increased puromycin integration signals started in 1 h post-H_2_O_2_-treated So-RNaseZover strain but almost the same signal intensities were found in the two strains after 3 h treatment (Fig. 6B). Accordingly, the bacterium recovered cellular So-RNaseZ content after 3 h treatment with H_2_O_2_ (Fig. 6A); this indicates that H_2_O_2_-caused translation suppression is related to the cellular So-RNaseZ levels.

Next, So-RNaseZ overexpression on bacterial growth under 0.2 and 0.5 mM H_2_O_2_ was assayed. Figure 6C shows that strain So-RNaseZover displayed a growth rate lower than that of strain pDL278RNaseZ-6×His even without H_2_O_2_ treatment, presumably because excessive increased translation could cause protein misfolding and aggregation. Treatment with 0.2 and 0.5 mM H_2_O_2_ resulted in significant growth decrease of the two strains within 3 h, by 9.27- and 20.69-fold for pDL278RNaseZ-6×His and 3.51- and 6.28-fold for So-RNaseZover strain, respectively. However, we found growth rates for strain So-RNaseZover 2.14- and 2.67-fold higher than those for pDL278RNaseZ-6×His upon 0.2 and 0.5 mM H_2_O_2_ treatment, respectively (Fig. 6C). This determines that So-RNaseZ overexpression partly rescues H_2_O_2_-arrested growth.

### So-RNaseZ oxidative degradation confers *S. oligofermentans* with oxidative adaptability.

To explore the physiological significance of So-RNaseZ degradation-mediated protein synthesis and growth suppression when bacteria encounter H_2_O_2_ stress, the higher H_2_O_2_ survivability was compared for the wild-type and So-RNaseZ-overexpressed *S. oligofermentans*. As 0.5 mM H_2_O_2_ treatment resulted in So-RNaseZ degradation to a lower content in pDL278RNaseZ-6×His than that in So-RNaseZover strain (Fig. 6A), these two strains were first subject to a 30-min treatment with 0.5 mM H_2_O_2_ and then challenged with 20 mM H_2_O_2_ for 10 min. The survival percentages were calculated by dividing the CFU of 20 mM H_2_O_2_-treated samples by those of the untreated samples. The results showed that So-RNaseZover strain exhibited a survival statistically significantly (Student’s *t* test, *P = *0.01) lower than that of the wild-type strain pDL278RNaseZ-6×His (61.36 ± 7.85% for So-RNaseZover and 83.14 ± 4.30% for pDL278RNaseZ-6×His). This indicates that So-RNaseZ oxidative degradation and mediated translational regulation provide the oxidative adaptability for *S. oligofermentans*.

## DISCUSSION

**FIG 7:**
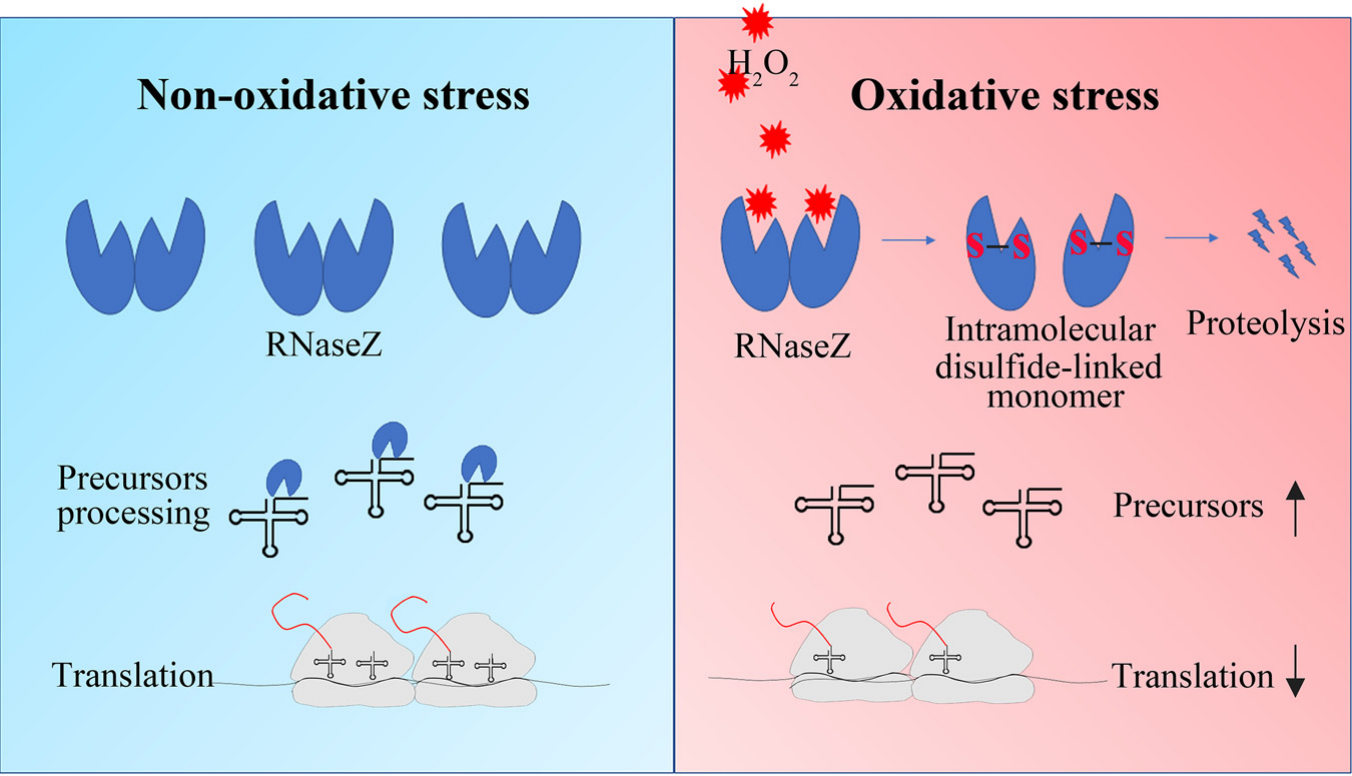
So-RNaseZ oxidative degradation impedes tRNA 3′ end maturation and results in slowing down of translation in streptococci. When *S. oligofermentans* is exposed to H_2_O_2_, So-RNaseZ (RNaseZ), the RNase specifically processing the tRNA 3′ end for maturation, is oxidized to form intramolecular Cys38-Cys149 linkages. The disulfide linkages substantially contributed to So-RNaseZ degradation. Decreased cellular So-RNaseZ levels result in the accumulation of tRNA precursors and, accordingly, suppression of translation and reduced protein (red curves) synthesis under H_2_O_2_ stress.

To cope with oxidative stress, organisms have developed diverse mechanisms (2–4). In addition to upregulating antioxidant genes, translation regulation provides an alternative mechanism in response to oxidative stress by reducing protein synthesis within minutes (8). Translation suppression under oxidative stress can be more significant for streptococci, as they lack catalase and cannot rapidly remove cellular H_2_O_2_. This study reports that oxidative degradation of So-RNaseZ impedes tRNA 3′ end maturation and, thus, slows down protein translation and growth of streptococci when exposed to H_2_O_2_, therefore revealing a new translation regulatory mechanism responding to oxidative stress in bacteria. As depicted in Figure 7, when streptococci are exposed to H_2_O_2_, So-RNaseZ, an RNase specifically processing the tRNA 3′ end for maturation, would be oxidized to form intramolecular Cys38-Cys149-linked aberrant conformations and was subject to proteolysis. The reduced cellular So-RNaseZ content results in the accumulation of tRNA precursors and, accordingly, suppression of the overall protein synthesis and growth of streptococci. Overexpression of So-RNaseZ elevates tRNA precursor processing and protein synthesis and partly rescues growth of *S. oligofermentans* under H_2_O_2_ stress. Therefore, this work revealed that So-RNaseZ, a streptococcal tRNAs 3′ end maturation enzyme, modulates the protein translation through posttranslational oxidative modification. Moreover, So-RNaseZ oxidative degradation-mediated translation repression offered oxidative adaptability to *S. oligofermentans*. Genomes of bacteria in the family of *Streptococcaceae* encode all CCA-less tRNAs, and their RNase Zs all possess Cys38 and Cys149 (Fig. S9), suggesting that the mechanism found in this study could be widely employed by catalase-negative streptococci and lactococci.

Streptococci are well known for producing, as well as tolerating, high concentrations of H_2_O_2_ (22, 23), so they could have evolved diverse strategies to cope with H_2_O_2_ stress (3, 24, 41, 42). Previously, we found that *S. oligofermentans* employs the H_2_O_2_-sensitive cysteine residues to sense H_2_O_2_ and regulate an inducible oxidative stress defense (28). Through cysteine oxidization, *S. oligofermentans* PerR derepresses expression of metal homeostasis-maintaining genes, such as *dpr*, which chelates excess cellular Fe^2+^ to avoid Fenton chemistry-mediated •OH production, and *mntABC*, which imports Mn^2+^ to decompose cellular H_2_O_2_ (28, 29). S. pneumoniae and *S. oligofermentans* also employ cysteine oxidization mechanisms to activate redox systems like thioredoxin to reduce oxidized proteins and to inactivate key metabolic proteins, such as glyceraldehyde 3-phosphate dehydrogenase, to decrease carbohydrate metabolism (22, 28). Interestingly, this study found that H_2_O_2_-sensitive cysteine oxidization of the tRNA processing endoribonuclease So-RNaseZ caused its degradation, thus leading to repression of protein synthesis and a growth pause of *S. oligofermentans* in response to oxidative stress. Moreover, So-RNaseZ oxidative degradation-mediated translation repression provides the oxidative stress adaptability for *S. oligofermentans*. This suggests that H_2_O_2_-sensitive cysteine oxidization could be a general strategy used by catalase-lacking streptococci in response to oxidative stress.

Cysteine is the amino acid residue most sensitive to H_2_O_2_ oxidation (43). Reversible cysteine thiol oxidizations, such as sulfenic acid (SOH) and disulfide bonds, usually function in the activation or suppression of redox regulatory and antioxidant proteins (44–46). Escherichia coli OxyR was the first identified archetype of thiol-based redox regulators in bacteria, which is activated by H_2_O_2_ oxidization-resulted intramolecular thiol-disulfide formation, and thereby induces the expression of antioxidant genes (5). So-RNaseZ contains two cysteine residues, Cys38 and Cys149, and this study found that H_2_O_2_ mediated Cys38-Cys149 and Cys149-Cys149 disulfide linkage formation in So-RNaseZ (Fig. 2; Fig. S3). Subsequent superimposition of a So-RNaseZ homology model over the monomeric crystal structure of B. subtilis RNase Z (PDB number 4GCW) showed that Cys38 and Cys149 were situated near the N terminus of the α1 helix and the β9 strand, respectively (Fig. 1B), and such locations may endow the two residues with H_2_O_2_ sensitivity, because cysteine residues near the N terminus of a helix are more likely to possess lower pK_a_ values (47). H_2_O_2_-oxidized So-RNaseZ disulfide linkages further resulted in intramolecular disulfide-linked monomer and oligomer formation (Fig. 2 and 3), which was then subject to rapid proteolysis (Fig. 3; Fig. S5). Similar findings were also reported in H_2_O_2_-mediated degradation of uracil-DNA glycosylase (UNG1) in human mitochondria (48) and proteolysis of H_2_O_2_-oxidized disulfide-linked metalloregulator MntR oligomers in *S. oligofermentans* (36). Notably, serine substitution of Cys38 and Cys149 reduced So-RNaseZ activity by approximately 8- and 4-fold, respectively (Fig. S10), probably because C38S mutation dramatically and C149S mutation slightly changed the protein conformation of So-RNaseZ (Fig. S6). Additionally, Cys38 and Cys149 are located near the Zn^2+^-binding photodiesterase (PDE) domain (Fig. 1B), and thus their mutation-caused conformational changes also might interrupt Zn^2+^ binding. Therefore, Cys38 and Cys149 play roles not only in mediating the oxidative degradation of So-RNaseZ under H_2_O_2_ stress but also in maintaining So-RNaseZ activity. C149S mutation-caused attenuated endoribonucleolytic activity of So-RNaseZ might explain the failure of So-RNaseZC149S to help alleviate translation and growth repression of *S. oligofermentans* under H_2_O_2_ stress (data not shown).

Previous studies have indicated that organisms, through maneuvering the cellular tRNAs, modulate translation elongation and translation outcomes under H_2_O_2_ stress (14, 49, 50). When challenged by H_2_O_2_, the eukaryotic tRNAs would be cleaved near the anticodon or at the 3′CCA termini (8, 17, 18), whereas the E. coli tRNAs are degraded swiftly, resulting in significantly lower translation elongation rates (19, 20). Distinctly, when exposed to H_2_O_2_, streptococci maneuver their tRNAs through degradation of oxidized So-RNaseZ, the endoribonuclease specifically for tRNA 3′ end maturation, as revealed by this work. The 3′ end trailers of bacterial tRNA precursors usually are processed through two pathways (51). The first of these is the 3′ exoribonucleolytic pathway, which functions through the action of RNase T or RNase PH processing CCA-encoding tRNAs (52). Alternatively, the endoribonucleolytic pathway can achieve this through RNase Z processing those tRNAs lacking a CCA motif (30). In contrast to E. coli, which carries all CCA motif-embedded tRNA genes, all 50 *S. oligofermentans* tRNA genes are CCA-less (Fig. S1A), and thus 3′ end maturation should be implemented exclusively by So-RNaseZ. Accordingly, H_2_O_2_-induced So-RNaseZ degradation resulted in the accumulation of tRNA precursors and decrease in the levels of mature tRNAs in H_2_O_2_-treated *S. oligofermentans*, while overexpression of So-RNaseZ restored the levels of precursors processing and mature tRNAs (Fig. 4). Thus, the 3′ end specific processing enzyme So-RNaseZ plays important roles in the swift modulation of cellular mature tRNA levels under H_2_O_2_ challenge, thus contributing to translation and growth repression of *S. oligofermentans* (Fig. 5 and 6).

In summary, *S. oligofermentans*, through H_2_O_2_-oxidizing cysteine residues, reduces the cellular So-RNaseZ abundance and tRNA 3′ end maturation and thus suppresses protein translation and growth. Notably, the So-RNaseZ abundance and the nascent protein synthesis, as indicated by puromycin integration, were similar in the statically and anaerobically grown early-to-mid-exponential-phase cells (Fig. S11A and B). This indicates that So-RNaseZ oxidative degradation-caused translation repression could be used by *S. oligofermentans* to cope with higher H_2_O_2_ challenge but not the lower levels of H_2_O_2_ generated during static growth. Indeed, So-RNaseZ appears to retain in 80 μM H_2_O_2_ but is degraded in >200 μM H_2_O_2_-treated *S. oligofermentans* (Fig. 3A and B). Therefore, this posttranslational modification-based translation regulatory strategy could be employed by *S. oligofermentans* to specifically cope with H_2_O_2_ stress (Fig. 5). Given that the periodical oral hygiene prophylaxis imposes oxidative stress, the oral inhabitant *S. oligofermentans* could benefit from So-RNaseZ oxidative degradation-endowed oxidative adaptability. Given that So-RNaseZ orthologs, in particular the Cys38 and Cys149 residues, are widely distributed within the genera of *Streptococcus* and *Lactococcus* of *Streptococcaceae*, whose genomes all encode non-CCA tRNAs and lack catalase (Fig. S9), this mechanism of RNase Z oxidative degradation-based tRNA maturation and translation regulation could be widely employed by catalase-lacking bacteria in response to oxidative stress.

## MATERIALS AND METHODS

### Experimental strains and culture conditions.

*S. oligofermentans* AS 1.3089 (53) and its derivative strains (Table S1) were grown in brain heart infusion (BHI) broth (BD Difco, Franklin Lakes, NJ, USA) statically as a standing culture or anaerobically under 100% N_2_. Fe^2+^-lacking chemically defined FMC medium was prepared as described by Terleckyj et al. (54) but omitting FeSO_4_. Escherichia coli DH5α was used for cloning and grown in Luria-Bertani (LB) broth at 37°C with shaking. When required, kanamycin (1 mg/ml) and spectinomycin (1 mg/ml) were used for selection of Streptococcus transformants, while ampicillin (100 μg/ml) and spectinomycin (250 μg/ml) were used to select E. coli transformants.

### Construction of genetic strains.

All primers used in this study are listed in Table S1. For the construction of the 6×His-tagged strain, the So*-rnaseZ* gene was amplified from the *S. oligofermentans* genome DNA using a pair of primers with a reverse primer carrying 6 histidine residues encoding sequence immediately upstream of the termination codon. Meanwhile, a DNA fragment of ∼600 bp immediately downstream of the termination codon of the So*-rnaseZ* gene was also amplified. Purified PCR products were digested by BamHI and ligated with a kanamycin resistance gene fragment released from pALH124 (55). The ligation mixtures were transformed into *S. oligofermentans* wild-type strain as described previously (56) to obtain strain RNaseZ-6×His. For construction of the So-RNaseZ complemented strain, the So*-rnaseZ*-6×His gene was amplified from the genome DNA of the RNaseZ-6×His strain and fused with the promoter fragment of an 8-gene operon composed of So*-rnaseZ* and 7 other genes (I872_05450 to I872_05485) by overlapping PCR. Then, this fusion fragment was integrated into vector pDL278 (37) by Gibson assembly to produce pDL278-So-RNaseZ-6×His. Next, Cys38 and Cys149 were mutated into serine using a site-directed gene mutagenesis kit (Beyotime Biotechnology Co., Shanghai, China). The correct pDL278-So-RNaseZ-6×His, -So-RNaseZ-C38S-6×His, and -So-RNaseZ-C149S-6×His plasmids were transformed into the wild-type strain to produce wild-type and cysteine-mutated So-RNaseZ ectopically expressing strains, respectively. Subsequently, the genomic So*-rnaseZ* copy was deleted using kanamycin-resistant gene substitution. For construction of the So-RNaseZover strain, a lactate dehydrogenase promoter fragment was fused with the So*-rnaseZ*-6×His gene and then was integrated into pDL278 at compatible EcoRI and SalI sites. The correct construct was transformed into the wild-type strain.

### Overexpression of So-RNaseZ-6×His protein.

A 930-bp DNA fragment containing the entire So*-rnaseZ* coding region was PCR amplified with primers listed in Table S1 and then was integrated into pET-28a (Novagen, Madison, WI, USA) by Gibson assembly (NEB, Beverly, MA, USA) to produce pET-28a-So-RNaseZ. Verified constructs were transformed into E. coli BL21(DE3) (Novagen). Positive transformants were grown at 37°C to an optical density at 600 nm (OD_600_) of 0.6 to 0.8, and 0.1 mM IPTG (Sigma-Aldrich, St. Louis, MO, USA) was added to the cultures to induce protein expression and the cultures were further incubated at 22°C overnight. Then, cells were collected by centrifugation, resuspended in binding buffer (20 mM Tris-HCl, 500 mM NaCl, 20 mM imidazole, 1 mM EDTA, and 1 mM DTT [pH 7.4]) and lysed by sonication for 45 min. The supernatant was filtered and applied to a Ni^2+^-charged chelating column (GE Healthcare, Piscataway, NJ, USA) previously equilibrated with binding buffer. Proteins were then eluted using elution buffer (20 mM Tris-HCl, 500 mM NaCl, 500 mM imidazole, and 1 mM DTT [pH 7.4]). The fractions with desired proteins were pooled and dialyzed against buffer containing 20 mM Tris-HCl, 150 mM NaCl, 1 mM DTT, and 1 mM EDTA three times. Purified So-RNaseZ-6×His protein was then stored in aliquots in 10% glycerol at –80°C until use.

### Nonreducing SDS-PAGE.

Five micrograms of So-RNaseZ-6×His protein was treated with or without various concentration of H_2_O_2_ for 30 min and with or without a subsequent reduction by 10 mM DTT for 1 h. Before electrophoresis, 40 mM NEM was added and samples remained in the dark for 30 min. These protein samples were then diluted in nonreducing SDS loading buffer (4× stock, 0.2 M Tris-HCl [pH 6.8], 40% glycerol, 8% SDS, and 0.4% bromophenol blue) and then separated on 12% SDS-PAGE gels.

### Redox Western blotting.

Cells were collected by centrifugation and resuspended in radio immunoprecipitation assay (RIPA) lysis buffer (50 mM Tris-HCl [pH 7.4], 150 mM NaCl, 1% Triton X-100, 1% sodium deoxycholate, 0.1% SDS, sodium orthovanadate, sodium fluoride, EDTA, and leupeptin) (Beyotime Biotechnology, Shanghai, China) with the addition of 40 mM NEM, 1 mM phenylmethylsulfonyl fluoride (PMSF), 10 mM EDTA, and 1 kilounit (KU)/ml catalase. Cells were sonicated on ice for 45 min and alkylated for 30 min in the dark, and then supernatants were collected by centrifugation. Protein concentrations were determined using a bicinchoninic acid (BCA) protein assay kit (ThermoFisher Scientific, Waltham, MA, USA). Protein samples were then diluted in nonreducing loading buffer (4× stock, 0.2 M Tris-HCl [pH 6.8], 40% glycerol, 8% SDS, and 0.4% bromphenol blue), separated by 10% SDS-PAGE, transferred onto nitrocellulose membranes, and probed with an anti-His tag antibody (Abmart Company, Shanghai, China) at a 2,000-fold dilution. Western blotting signals were detected using Amersham ECL prime Western blot detection reagent (GE Healthcare).

### *In vitro* transcription.

*In vitro* transcription assays were performed using the MEGAshortscript T7 transcriptional kit (ThermoFisher Scientific) per the manufacturer’s protocol. Various lengths of tRNA precursor fragments were amplified from the *S. oligofermentans* genome DNA using pairs of primers, with the 5′ end primer containing a T7 RNA polymerase promoter sequence (Table S1). PCR products were gel purified and used as the templates. The reaction mixture (20 μl) contained 2 μl 10× T7 reaction buffer, 2 μl T7 enzyme mix, 75 mM (each) ATP, CTP, GTP, and UTP, 400 mM GMP, 40 U RNase inhibitor (Promega, Madison, WI, USA), and 2 μg of template. Transcription reactions were performed at 37°C for 4 h. Template DNA and unincorporated nucleotides were removed using TURBO DNase (Promega) and G50 spin columns (Amersham), respectively. After denaturation at 70°C for 10 min, the products were immediately put on ice to open transcript secondary structures. Transcript purity was examined by 10% urea-PAGE gels that were stained with 3,8-diamino-5-ethyl-6-phenylphenanthridin-5-ium bromide (EB), and products were preserved at –80°C until use.

### So-RNaseZ activity assay.

So-RNaseZ nucleolytic activity was assayed as described previously (30) with slight modification. In brief, the 10 μl reaction buffer contained 40 mM Tris-HCl (pH 8.4) and 2 mM (each) MgCl_2_, KCl, and dithiothreitol (DTT). Fifty nanograms of tRNA precursor were used per assay, this nucleolytic assay was initiated by the addition of various concentrations of purified So-RNaseZ, and the reaction mixture was incubated at 37°C for 30 min. Then, 1 μl of protease K was added and the reactions were maintained at room temperature for 15 min to digest So-RNaseZ. Each 10 μl reaction was mixed with 10 μl loading buffer (2× stock, 98% formamide, 5 mM EDTA, 0.025% SDS, and 0.025% bromophenol blue). After denaturation at 70°C for 10 min, the products were immediately put on ice to open RNA secondary structures and then run on 10% urea-PAGE gels and stained with SYBR gold nucleic acid gel stain (ThermoFisher Scientific).

### Northern blotting.

RNA samples were separated on 10% urea-PAGE gels, transferred to positively charged nylon membranes (GE Healthcare), and cross-linked using a GS Gene Linker UV chamber. The membranes were prehybridized in buffer (5× SSC [1× SSC is 0.15 M NaCl plus 0.015 M sodium citrate], 5× Denhardt’s, 50% [vol/vol] deionized formamide, 0.5% [mass/vol] SDS, and 200 μg ml^−1^ salmon sperm DNA) for 4 h and then hybridized with biotin-labeled probes (Table S1) at 42°C overnight and detected by chemiluminescent nucleic acid detection module kit (ThermoFisher Scientific).

### Western blotting.

*S. oligofermentans* cells were first sonicated in RIPA lysis buffer (Beyotime Biotechnology) containing 1 mM PMSF. Cell lysates were then collected by centrifugation. Same amounts of proteins were separated by SDS-PAGE and hybridized with horseradish peroxidase (HRP)-conjugated anti-His tag monoclonal antibody at a 1:2,000 dilution (Abmart, Shanghai, China). Anti-puromycin monoclonal antibody at a 1:10,000 dilution (Millipore Company, Darmstadt, Germany) and HRP-conjugated anti-mice secondary antibody (Abmart) at a 1:2,000 dilution were used to detect puromycin-integrated proteins. Western blotting signals were detected using the Amersham ECL prime Western blot detection reagent (GE Healthcare). Intensities of target bands were quantified using Image J.

### 5′ and 3′ RACEs.

5′ and 3′ RACEs were performed as described previously (57) with slight modifications. Briefly, So-RNaseZ-digested products of tRNA precursor transcripts were purified on 10% urea-PAGE gels using a ZR small-RNA PAGE recovery kit (Zymo Research, Irvine, CA, USA), and then the 5′ and 3′ ends were ligated with 50 pmol of universal microRNA (miRNA)-cloning linker 5′-CAGACUGGAUCCGUCCUC-App-3′ and 5′-AppCUGUAGGCACCAUCAAU-ddC-3′ (synthesized by Integrated DNA, Coralville, IA, USA), respectively, with the addition of 20 U of T4 RNA ligase (Ambion, Austin, TX, USA) in the presence of 40 U of RNasin-Plus. The reaction mixtures were incubated at 16°C for 16 h, and the 5′- and 3′-linker-ligated RNAs were recovered by isopropanol precipitation and then mixed with 2 pmol of random primer and 100 pmol of 3′ RACE RT primer (5′-ATTGATGGTGCCTACAG-3′; complementary to the universal miRNA cloning linker), respectively. Full-length cDNAs were synthesized by using 200 U SuperScript III reverse transcriptase (Invitrogen, Carlsbad, CA, USA). The 5′ and 3′ end-containing cDNA fragments were next amplified using nested PCR using primers listed in Table S1. PCR products were recovered from a 2% (wt/vol) agarose gel and then TA-cloned into pMD19-T (TaKaRa, Dalian, China), followed by colony PCR and sequencing.

### LC-MS/MS identification of disulfide-linked peptides of the recombinant So-RNaseZ protein.

Recombinant 6×His-tagged So-RNaseZ protein was treated with 500 μM H_2_O_2_ and then incubated with 55 mM iodoacetamide (IAM) in the dark at 37°C for 1 h before separation on 12% nonreducing SDS-PAGE gels. After being stained with Coomassie blue G-250, protein bands were sliced and cut into pieces. Gel pieces were washed twice with MS-grade water, alkylated with 55 mM iodoacetamide (IAM) at 37°C for 1 h, and then digested using sequencing grade modified trypsin (Promega, Fitchburg, WI, USA) in 50 mM NH_4_HCO_3_ (pH 8.0) at 37°C overnight. Digested products were extracted twice with 1% formic acid in 50% acetonitrile aqueous solution and dried to reduce volume using a Speedvac. For LC-MS/MS analysis, peptides were separated using a 65-min gradient elution at a flow rate of 0.250 ml/min with the EASY-nLC integrated nano-high-pressure liquid chromatography (HPLC) system (ThermoFisher Scientific), which was directly interfaced with a Thermo Q-Extractive mass spectrometer. The analytical column was a homemade fused silica capillary column (75 mm internal diameter, 150 mm length; Upchurch Scientific, Oak Harbor, WA, USA) packed with C_18_ resin (300 Å, 5 μm; Varian, Palo Alto, CA, USA). Mobile phase A consisted of 0.1% formic acid, and mobile phase B consisted of 100% acetonitrile and 0.1% formic acid. The Q-Extractive mass spectrometer was operated in the data-dependent acquisition mode using Xcalibur 3.0 software. A single full-scan mass spectrum in the Orbitrap (300 to 1,800 *m/z*, 70,000 resolution) followed by 20 data-dependent MS/MS scans in the ion trap at 27% normalized collision energy. Each mass spectrum was searched against the So-RNaseZ protein sequence (NCBI accession no.: AGK71184.1) using the SEQUEST searching engine of Proteome Discoverer software (v1.4). Peptide fragments with disulfide linkage were analyzed by PMi-Byonic software (Protein Matrix Inc., CA, USA).
