# Supplementary material for: RNase Z Oxidative Degradation Impedes tRNA Maturation and is Involved in Streptococcal Translation Regulation in Response to Oxidative Stress
Source: Microbiol Spectr. 2021 Oct 27;9(2):e01167-21. doi: 10.1128/Spectrum.01167-21 (PMC8549757; doi:10.1128/Spectrum.01167-21)
Supplement: SUPPLEMENTAL FILE 1 — Supplemental material. Download SPECTRUM01167-21_Supp_1_seq9.pdf, PDF file, 1.7 MB [file spectrum01167-21_supp_1_seq9.pdf]

**Supplementary Information for**

# RNase Z oxidative degradation impedes tRNA maturation and is involved in streptococcal translation regulation in response to oxidative stress

Yuzhu Dong[a,b,1], Huichun Tong[a,b,1,*], Qingqing Hu[a,b], Xiuzhu Dong[a,b,*]

[a]State Key Laboratory of Microbial Resources, Institute of Microbiology, Chinese Academy of Sciences, Beijing 100101, China

[b]University of Chinese Academy of Sciences, Beijing 100049, China

**This file contains:**

Figures S1 to S11

Table S1

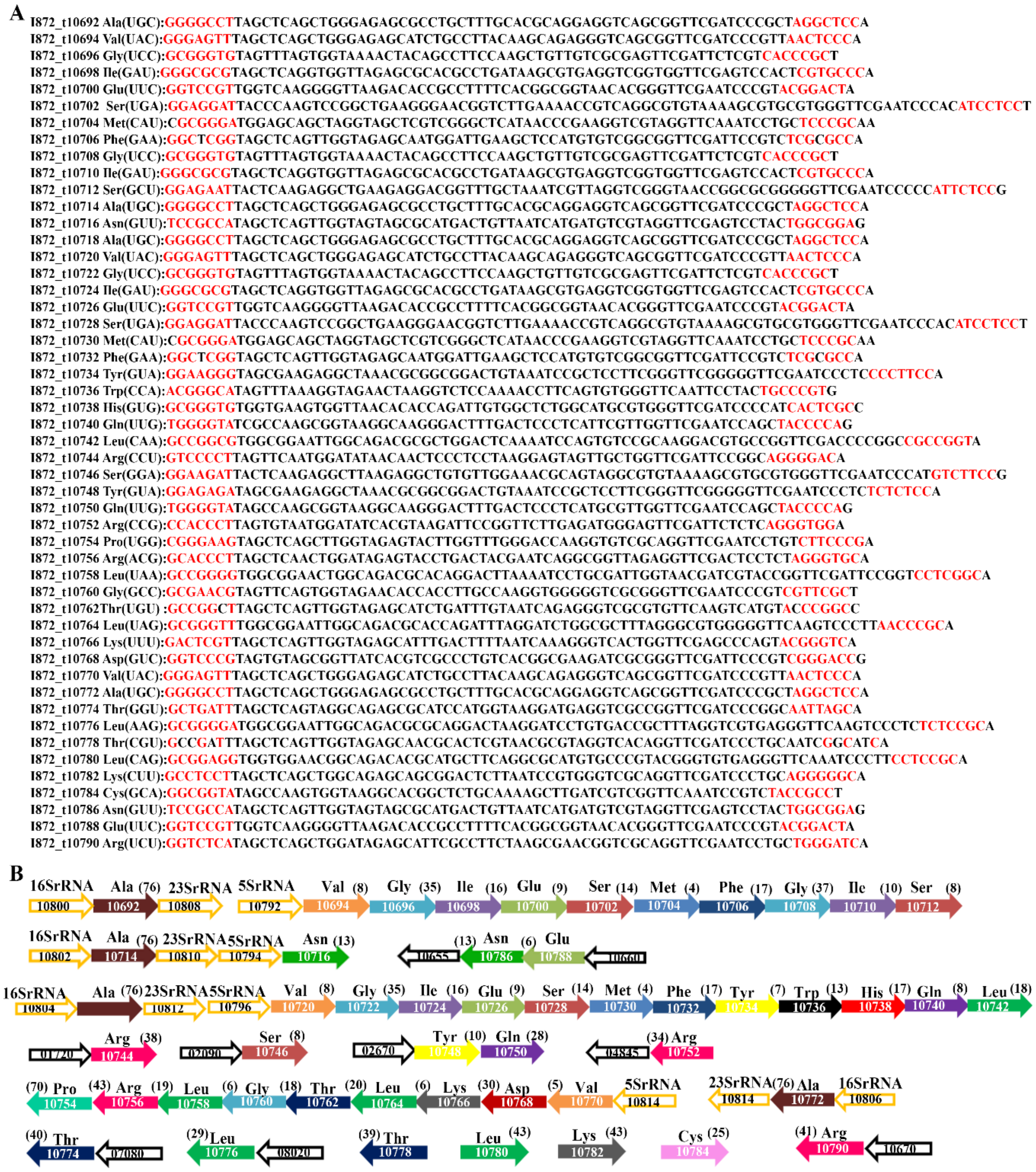

**Fig. S1. Sequences (A) and the genomic organizations (B) of the 50 *S. oligofermentans* tRNA genes.** A. Sequences of the 50 tRNA genes of *S. oligofermentans* are retrieved from KEGG database, and the accession numbers and amino acid trigrams are provided. Nucleotides in parentheses are the anticodons and those in red are reversely complemented to form the acceptor stem. The last one in each

sequence is the discriminator nucleotide. B. Schematics show the genomic organizations of the *S. oligofermentans* tRNA genes (solid bullets) with other genes (empty bullets). The amino acid trigram represents its carrier tRNA gene with the accession numbers shown. Number inside parenthesis at the 3’ end of each tRNA gene represents the 3' trailer length of the tRNA precursor. The tRNA genes shown in the same colored bullets carry the same amino acid.

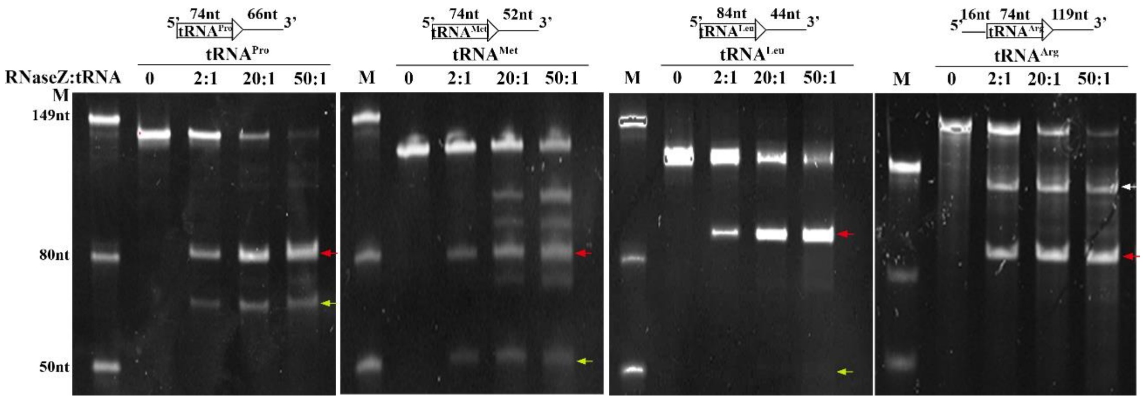


**Fig. S2. Urea-PAGE analysis of So-RNaseZ cleaving four tRNA precursors.** The 140 nt tRNA$^{Pro}$ (I872_t10754), 126 nt tRNA$^{Met}$ (I872_10730), 128 nt tRNA$^{Leu}$ (I872_t10780) and 209 nt tRNA$^{Arg}$ (I872_t10790) precursor fragments were obtained by *in vitro* transcription, and 50 ng of each tRNA precursor was used as the nucleolytic substrates. Nucleolytic assay was initiated by addition of So-RNaseZ protein in a gradient increased molar ratio as indicated, and incubated at 37ºC for 30 min as described in "Materials and Methods". Nucleolytic products were separated on 10% Urea PAGE gel. Schematics at top show the lengths of 5' extension, tRNA gene and 3' trailer in each tRNA precursor. Red and yellow arrows indicate the cleaving products comprising of tRNA gene or 5' extension and tRNA gene for tRNA$^{Arg}$ precursor, and 3' trailer, respectively. M, RNA molecular weight marker is shown at the left.

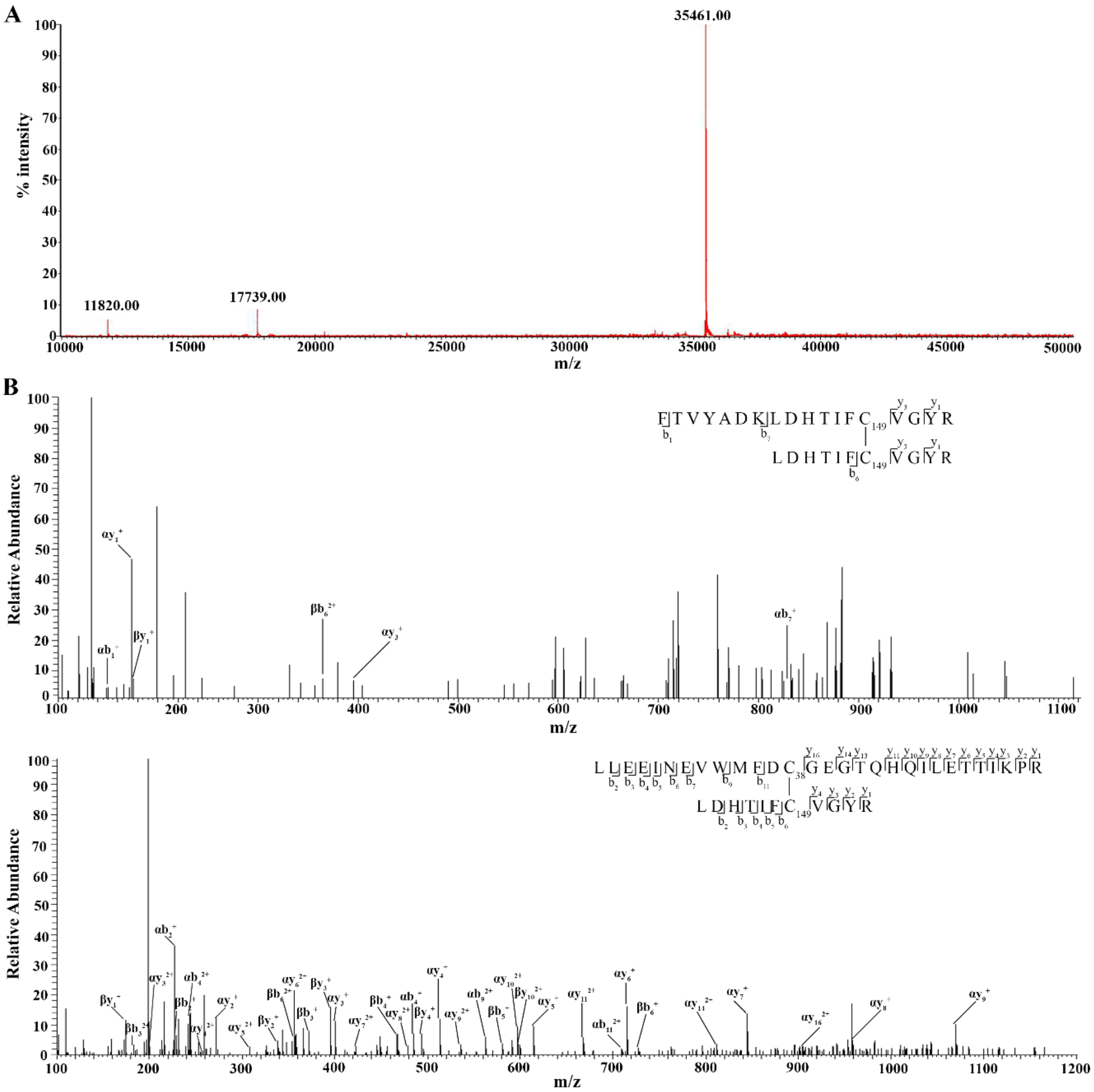


**Fig. S3. Mass spectrometry analysis of So-RNaseZ protein and the disulfide linkages.** A. Ten microgram recombinant So-RNase Z protein in 500 μL PBS was reduced with 10 mM DTT, and the molecular weight was identified by MALDI-TOF analysis. One main peak with molecular mass of 35.461 kDa was identified corresponding to the predicted molecular mass of recombinant So-RNaseZ plus 6 Histidine tag (35.15 kDa). B. The So-RNaseZ protein bands named “o”, “dd” and “id” in Figure 2A were sliced and trypsin digested as described in “Materials and Methods”,

and then subject to LC-MS/MS identification. The upper panel shows the LC-MS/MS spectrum of a 4-charged peptide fragment with the precursor mass of 3468.7258 that was identified as Cys149-Cys149 linked peptide as shown in insert. The lower panel shows the LC-MS/MS spectrum of a 6-charged peptide fragment with precursor mass of 4766.3212 that was identified as an intra-molecular Cys38-Cys149 linked peptide (insert).

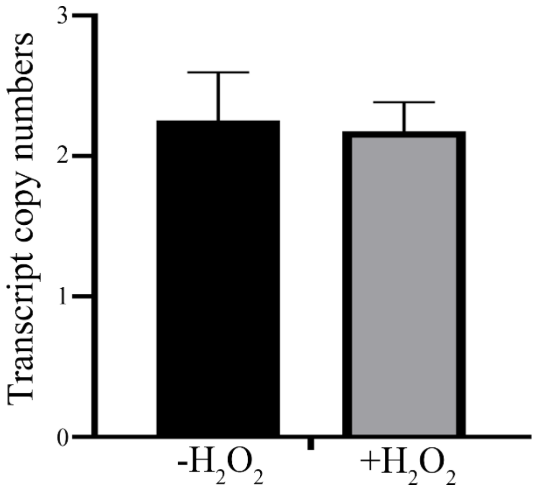


**Fig. S4. qPCR determines the So-*rnaseZ* transcript levels in anaerobically grown *S. oligofermentans* treated (+) with or without (-) $H_2O_2$.** Total RNAs were extracted from the mid-exponential cells using TRIzol reagent. After quality confirmation on 1% agarose gel, cDNAs were generated from 2 μg of total RNA with random primers using Moloney murine leukemia virus reverse transcriptase, and then qPCR was implemented. Experiments were repeated three times on triplicate samples. Transcript copies were calculated as per 1000 16S rRNA copies, and the averages ± SD of three independent experiments are shown.

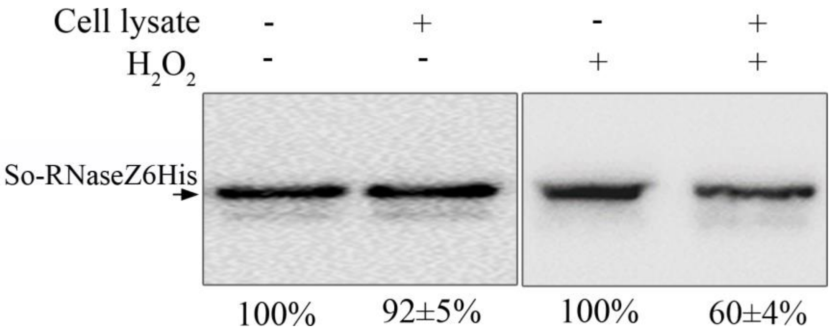


**Fig. S5. Determination of $H_2O_2$ oxidized So-RNaseZ degradation by the *S. oligofermentans* cell lysate.** The recombinant So-RNaseZ6His protein (10 µg) was first reduced with 10 mM DTT at 37ºC for 1 h, and then super-filtrated to remove DTT. An aliquot of So-RNaseZ protein was treated with 500 µM $H_2O_2$ for 30 min, and leaving another aliquot untreated as a control. After 30 min co-incubation with or without the cell lysate of *S. oligofermentans* at 37°C, So-RNaseZ6His protein was run on 12% SDS-PAGE gel and the abundance was determined by western blot using the anti-His antibody. Band intensities were measured using Image J, and expressed as the percentiles of the protein treated with and without $H_2O_2$ among their respective abundance in non-cell lysate treatments. Experiments were repeated three times, and the averages ± SD of three experiments are shown beneath the representative gel.

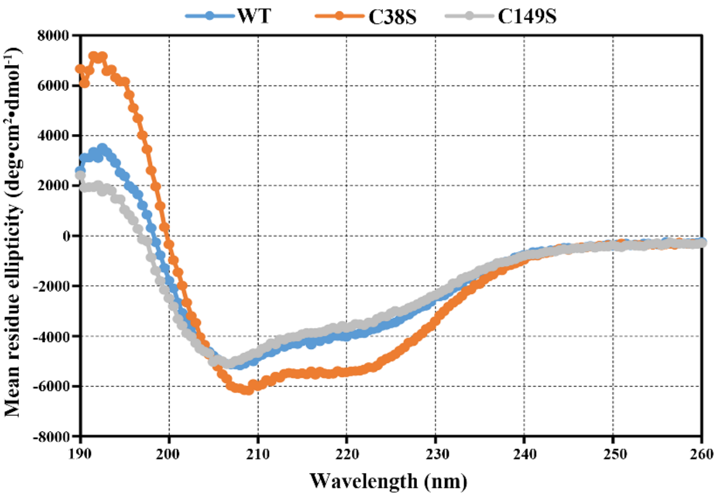


| | Helix1 | Helix2 | Strand1 | Strand2 | Turns | Unordered | Total |
|---|---|---|---|---|---|---|---|
| WT | 0.037 | 0.097 | 0.204 | 0.124 | 0.131 | 0.408 | 1.001 |
| C38S | 0.065 | 0.102 | 0.198 | 0.117 | 0.128 | 0.390 | 1 |
| C149S | 0.031 | 0.096 | 0.203 | 0.124 | 0.132 | 0.414 | 1 |

**Fig. S6. Circular dichroism (CD) spectra of the wild-type and cysteine mutated So-RNaseZ.** The So-RNaseZ6His (WT) Cys38 and Cys149 were each mutated into serine to construct So-RNaseZC38S6His (C38S) and So-RNaseZC149S6His (C149S), respectively. The three proteins were diluted into 200 µl CD buffer (10 mM $K_3PO_4$, 100 mM NaF, pH 7.5) to make a final concentration of 5 µM. CD spectra were collected in 0.1 cm quartz cuvettes from wave length of 190 to 260 nm at 0.5 nm interval with a 60 nm/min scan rate at Chirascan circular dichroism spectrometer (Applied photophysics Ltd) under a constant nitrogen purge. Data were acquired at 25 °C, and processed using the pro-Data Viewer 4.2.15 (Spectra Manager software) (Jasco, USA). Each spectrum represents the average of three scans of a sample. Protein secondary structures were analyzed via website Dichroweb (http://dichroweb.cryst.bbk.ac.uk), the output results were shown below the figure.

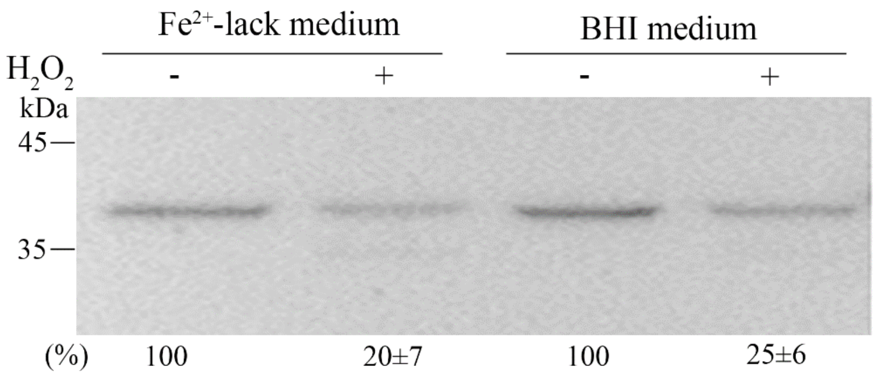


**Fig. S7. Assay of $H_2O_2$ or Fenton chemistry-generated hydroxyl radical caused the cellular So-RNaseZ degradation.** The *S. oligofermentans* RNaseZ-6His strain was anaerobically grown in $Fe^{2+}$-lack chemically defined FMC medium and BHI broth, respectively. Until $OD_{600}$ of ~ 0.3-0.4, an aliquot of the culture was 30 min treated with 500 μM $H_2O_2$, and leaving another aliquot untreated. Cells were then sonicated and the lysates were run on 12% SDS-PAGE gels. The cellular So-RNaseZ abundance was examined by western blot using anti-His antibody. Band intensities were measured using Image J, and expressed as the percentiles of the So-RNaseZ protein in the bacterium grown in the corresponding medium not treated with $H_2O_2$. Molecular weight marker is shown at the left. Experiments were repeated three times, and the averages ± SD of three experiments are shown beneath the representative gel.

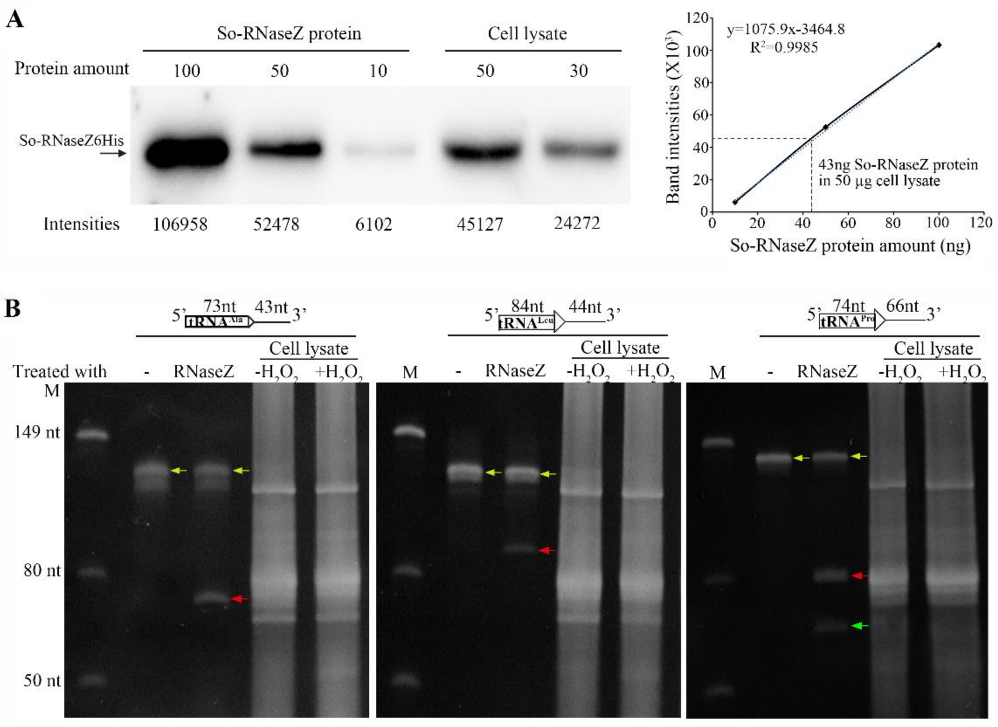


**Fig. S8. Determination of tRNA precursor degradation by *S. oligofermentans* cell lysates.** A. Western blot assayed the cellular So-RNaseZ protein content. Various amounts of purified So-RNaseZ protein (ng) and cell lysates (μg) of the RNaseZ-6His strain were run on 12% SDS-PAGE gel and So-RNaseZ protein was detected using anti-His antibody (left panel). Band intensities were measured using Image J. Band intensities of purified So-RNaseZ protein were plotted against So-RNaseZ protein amount (right panel). Referenced to the linear regression equation, 43 ng of So-RNaseZ protein was calculated in 50 μg cell lysate. B. The 116 nt $tRNA^{Ala}$ (I872_t10692), 128 nt $tRNA^{Leu}$ (I872_t10780) and 140 nt $tRNA^{Pro}$ (I872_t10754) precursor fragments were obtained by *in vitro* transcription. Four aliquots of 50 ng of each tRNA precursor were prepared; one aliquot was untreated as a control, two aliquots were incubated with 10

μg cell lysate of anaerobically cultured *S. oligofermentans* that were 30 min treated with (+$H_2O_2$) or without 0.5 mM $H_2O_2$ (-$H_2O_2$), respectively, and the other one was incubated with 15 ng So-RNaseZ protein (RNaseZ), by referenced to the So-RNaseZ abundance in 10 μg cell lysate estimated in A. After 30 min incubation, the samples were separated on 10% Urea PAGE gel and stained with SYBR Gold Nucleic Acid Gel Stain. Schematics at top show the lengths of the tRNA gene and 3' trailer in each precursor. Yellow arrow indicates the tRNA precursors, red and green arrows indicate the cleaving products comprising of tRNA gene and 3' trailer, respectively. M, RNA molecular weight marker with length shown at the gel left.

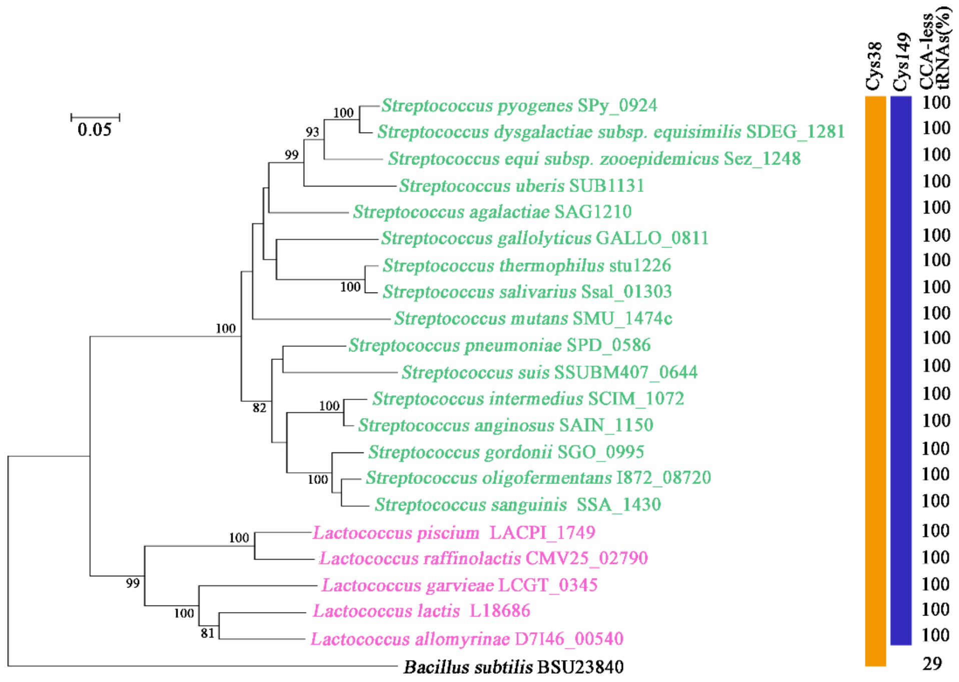


**Fig. S9. Phylogenetic analysis of the So-RNaseZ orthologs and its Cys38/Cys149 in Streptococcaceae that encode CCA-less tRNAs**. Sequences of So-RNaseZ protein and its orthologs from all species of Streptococcaceae were obtained from KEGG database, and aligned using ClustalW. The phylogenetic tree was constructed using the Neighbor-joining method with bootstrap values of 1000 replicates. The bar of 0.05 represents the evolution distance. Orange and blue bars at right indicate the presence of Cys38 and Cys149 in the RNase Z orthologs, respectively; and numbers represent the percentiles of CCA-less tRNAs in the respective species.

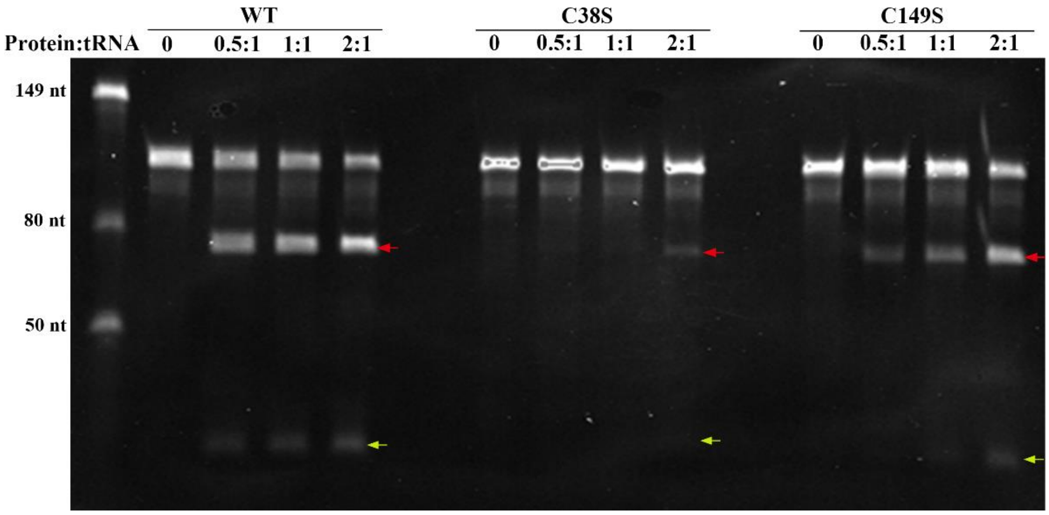


**Fig. S10. Comparison of the wild-type and cysteine mutated So-RNaseZ activities.** The 104 nt tRNA$^{Arg}$ (I872_t10790) precursor fragment, comprising of 74 nt mature tRNA$^{Arg}$ and 30 nt 3’ trailer, was obtained by *in vitro* transcription, and 50 ng was used as the nucleolytic substrates. Nucleolytic assay was initiated by addition of a gradient of increasing molar ratio of wild-type So-RNaseZ6His protein (WT) and Cys38 and Cys149 serine substituted So-RNaseZC38S6His (C38S) and So-RNaseZC149S6His (C149S), respectively, and incubated at 37ºC for 30 min as described in “Materials and Methods”. Nucleolytic products were separated on 10% Urea PAGE gel. Red and yellow arrows indicate the cleaving products of mature tRNA gene and 3' trailer, respectively. RNA molecular weight marker is shown at the gel left.

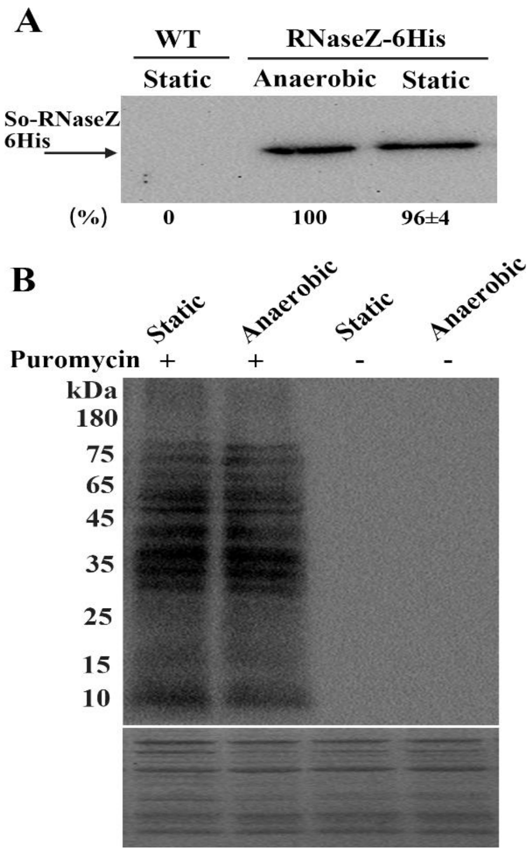


**Fig. S11. Comparison of So-RNaseZ abundance (A) and protein synthesis (B) of the statically and anaerobically grown *S. oligofermentans*.** A. Western blot assayed the So-RNaseZ abundance in RNaseZ-6His cells. Early mid-exponential cells of statically or anaerobically grown RNaseZ-6His were collected and lysed. Same amounts of total cell protein were loaded on 12% SDS-PAGE gels, and the So-RNaseZ abundance was examined by western blot using anti-His antibody. Cell lysate from statically grown wild-type (WT) cells was included as a control. Western blot signal intensities of the So-RNaseZ protein were measured using Image J, and expressed as the percentages of anaerobic culture. Triplicate experiments were performed, and averages ± SD are shown below one representative image. B. Strain RNaseZ-6His was cultured as described in A, and puromycin was added 10 min before cell collection.

Western blot assayed puromycin integration using the anti-puromycin antibody (upper panel), and same contents of total proteins were run on 12% SDS-PAGE gels and stained with Coomassie brilliant blue (lower panel). Molecular weight marker is shown at each gel left. Triplicate experiments were performed, and one representative result is shown.

**Table S1. Strains, plasmids and primers used in this study**[*]

| Strain, plasmid or primer | Characteristics and description | Reference or sources |
|---|---|---|
| Strain | | |
| *E. coli* | | |
| DH5α | *supE44 lacU169* (80 *lacZ*M15) *hsdR17 recA1 endA1 gyrA96 thi-1 relA1 luxS* | TransGen Biotech, Beijing, China |
| BL21 (DE3) | F- *ompT hsdS* (rBB - mB -) gal dcm (DE3) | TransGen Biotech, Beijing, China |
| *S. oligofermentans* | | |
| Wild-type | WT, Kan$^{s}$ Sp$^{s}$ | 1 |
| RNaseZ-6His | WT RNaseZ::6His; Kan$^{r}$; wild-type with RNaseZ fused with 6×His tag at C-terminus | This study |
| So-RNaseZcom | WT RNaseZ::Kan; pDL278-SoRNaseZ6His; Kan$^{r}$; Sp$^{r}$; wild-type with So-*rnaseZ* deletion and carrying pDL278-SoRNaseZ6His plasmid | This study |
| So-RNaseZC38Scom | WT RNaseZ::Kan; pDL278-SoRNaseZC38S6His; Kan$^{r}$; Sp$^{r}$; wild-type with So-*rnaseZ* deletion and carrying pDL278-SoRNaseZC38S6His plasmid | This study |
| So-RNaseZC149Scom | WT RNaseZ::Kan; pDL278-SoRNaseZC149S6His; Kan$^{r}$; Sp$^{r}$; wild-type with So-*rnaseZ* deletion and carrying pDL278-SoRNaseZC149S6His plasmid | This study |
| So-RNaseZover | WT pDL278-P*ldh*-SoRNaseZ6His; Sp$^{r}$; wild-type harboring pDL278 plasmid with So-RNaseZ6His driven by lactate dehydrogenase promoter | This study |
| pDL278RNaseZ-6His | WT RNaseZ::6His; Kan$^{r}$; RNaseZ-6His strain carrying pDL278 plasmid | This study |
| **Plasmid** | | |
| pALH124 | Kan$^{r}$ | 2 |
| pDL278 | Sp$^{r}$ | 3 |

| | | |
|---|---|---|
| pDL278-SoRNaseZ6His | pDL278 carrying SoRNaseZ6His; $Sp^r$ | This study |
| pDL278-SoRNaseZC38S6His | pDL278 carrying SoRNaseZC38S6His; $Sp^r$ | This study |
| pDL278-SoRNaseZC149S6His | pDL278 carrying SoRNaseZC149S6His; $Sp^r$ | This study |
| **Primer** | **Sequence (5'-3')** | **Purpose** |
| RNaseZ-6His upF | CTGGTTCCCGTCTGCCTTATC | RNaseZ-6His construction |
| RNaseZ-6His upR | CTAA*GGATCC*CTAGTGGTGGTGGTGGTGTAACTCCACCTCTTCCAAG | RNaseZ-6His construction |
| RNaseZ-6His dnF | CTAA*GGATCC*TTTTCCAATTTAGGACAGTTTC | RNaseZ-6His construction |
| RNaseZ-6His dnR | GCTCCTTCCAACCAAAATCAGTCGG | RNaseZ-6His construction |
| GipDL278F | CACCACCACTAGTCTAGAGTCGACCTGCAGG | So-RNaseZcom construction |
| GipDL278R | CAGAAAAGGAGAAATCACCTGGCCGTCGTTTTAC | So-RNaseZcom construction |
| PoperonF | CGACGGCCAGGTGATTTCTCCTTTTCTGAACTTTTA | So-RNaseZcom construction |
| PoperonR | GTAATTGCATCTTTTTTCCTTGAAAAAATTTTAATTTTTC | So-RNaseZcom construction |
| *rnz*-comF | AGGAAAAAAGATGCAATTACAATTTTTAGGAACCG | So-RNaseZcom construction |
| *rnz*-comR | GGTCGACTCTAGACTAGTGGTGGTGGTGGTGGTGTAACTCCACCTCTTC | So-RNaseZcom construction |
| *rnz*-deleteupF | GATTTGGCCCTGAAATACGGCGG | So-RNaseZcom construction |
| *rnz*-deleteupR | CCTA*GGATCC*GTAATTGCATGTTATTCTTTCTAAATG | So-RNaseZcom construction |
| *rnz*-deletedownF | GGA*GGATCC*GGAGTTATAGTTTTCCAATTTAG | So-RNaseZcom construction |
| *rnz*-deletedownR | CAGTCGGTCATGGGGCAAGAGC | So-RNaseZcom construction |
| *rnz*C38SF | GGTCTGGATGTTTGATTCCGGTGAAGGTACCCAG | So-RNaseZC38Scom construction |
| *rnz*C38SR | CTGGGTACCTTCACCGGAATCAAACATCCAGACC | So-RNaseZC38Scom construction |
| *rnz*C149SF | GCTAGACCACACGATTTTCTCTGTGGGTTATCGGGTCATGC | So-RNaseZC149Sco |

| | | |
|---|---|---|
| | | m construction |
| *rnz*C149SR | GCATGACCCGATAACCCACAGAGAAAATC GTGTGGTCTAGC | So-RNaseZC149Scom construction |
| PLDHF | CCG*GAATTC*TAAAAAATGCCTCGTTTCAAT ATTAAATC | So-RNaseZover construction |
| PLDHR | CCGGTTCCTAAAAATTGTAATTGCATTTCT AAACATCTCCTTATTATTTTTAG | So-RNaseZover construction |
| Pldh-*rnz*overF | CTAAAAATAATAAGGAGATGTTTAGAAAT GCAATTACAATTTTTAGGAACCGG | So-RNaseZover construction |
| Pldh-*rnz*overR | ACGC*GTCGAC*CTAGTGGTGGTGGTGGTGG TGTAACTCCACCTCTTCC | So-RNaseZover construction |
| TransArgF | TAATACGACTCACTATAGGGAGAATGAAA AGGTATCGGTCTC | *In vitro* transcription for RNaseZ activity |
| TransArgR | CTTACAAGATCTATTATAAATAAAATTTC | *In vitro* transcription for RNaseZ activity |
| TransAlaF | TAATACGACTCACTATAGGGGGGCCTTA GCTCAG | *In vitro* transcription for RNaseZ activity and Northern blot |
| TransAlaR | CAATTTTCAATGGACAAGTTTAGAATAGTC | In vitro transcription for RNaseZ activity |
| Northern AlaR | GTTTATTTTCTTGTTACTATTTGATATAGTT ATTC | In vitro transcription for Northern blot |
| TransProF | TAATACGACTCACTATAGGGCGGGAAGTA GCTCAG | In vitro transcription for RNaseZ activity |
| TransProR | CAAAAAAACGGCTCCAGCCGTTATTTAAA TAC | In vitro transcription for RNaseZ activity |
| TransLeuF | TAATACGACTCACTATAGGGGCGGAGGTG GTGGAACGG | In vitro transcription for RNaseZ activity |
| TransLeuR | CAAAAGAAAAACTCTTGTCAACTTG | In vitro transcription for RNaseZ activity |
| TransMetF | TAATACGACTCACTATAGGGCGCGGGATG GAGCAGCTAG | In vitro transcription for RNaseZ activity |

| | | |
|---|---|---|
| TransMetR | GACACATGGAGCTTCAATCC | In vitro transcription for RNaseZ activity |
| Ala probe | 5’-biotin-CGAACCGCTGACCTCCTGCGTGCAAAGCAGGCGCTCTCCCAG | Mature tRNA$^{Ala}$ Northern blot |
| Pro probe | 5’-biotin-CCTGCGACACCTTGGTCCCAAACCAAGTACTCTACCAAGCTGAGCTACTTCCCG | Mature tRNA$^{Pro}$ Northern blot |
| Leu probe | 5’-biotin-GATTTGAACCCTCACACCCGTACGGGCACATGCGCCTGAAGCATGCGTGTCT | Mature tRNA$^{Leu}$ Northern blot |
| pretRNA$^{Ala}$ probe | 5’-biotin-CTATTTGATATAGTTATTCAATTTTCAATGGACAAGTTTAGAATAG | tRNA$^{Ala}$ precursor Northern blot |
| pretRNA$^{Pro}$ probe | 5’-biotin-AAAAAAACGGCTCCAGCCGTTATTTAAATACAGTAATGATTAGAAAATTCTAAACCAACAATATA | tRNA$^{Pro}$ precursor Northern blot |
| pretRNA$^{Leu}$ probe | 5’-biotin-CATAATTTTTCAAAAGAAAAACTCTTGTCAACTTGGACAAGAGTTCCTGTTCGA | tRNA$^{Leu}$ precursor Northern blot |
| 5S rRNA probe | 5’-biotin-CACAGGGGGCAACCCCCAACTACTTCCGGCGTTCTAGGGCTTAACTTCTGTGTTCGGCATGGGTACAGGTGTAT | 5S rRNA Northern blot |
| Ala3RACEF | GGGGCCTTAGCTCAGCTGGG | tRNA$^{Ala}$ 3’ race |
| Ala3RACER | ATTGATGGTGCCTACAG | tRNA$^{Ala}$ 3’ race |
| Ala5RACEF | CAGACTGGATCCGTCCTC | tRNA$^{Ala}$ 5’ race |
| Ala5RACER | CAATTTTCAATGGACAAGTTTAGAATAGTC | tRNA$^{Ala}$ 5’ race |

*: Kan, kanamycin; Sp, spectinomycin; r, resistant; s, sensitive.

Italic nucleotide bases indicate restriction enzyme digestion sites.

## References


1. Tong H, Gao X, Dong X. 2003. *Streptococcus oligofermentans sp. nov.*, a novel oral isolate from caries-free humans. *Int J Syst Evol Microbiol* 53:1101-1104.
2. Liu Y, Zeng L, Burne RA. 2009. AguR is required for induction of the *Streptococcus mutans* agmatine deiminase system by low pH and agmatine. *Appl Environ Microbiol* 75:2629-2637.
3. LeBlanc DJ, Lee LN, Abu-Al-Jaibat A. 1992. Molecular, genetic, and functional analysis of the basic replicon of pVA380-1, a plasmid of oral streptococcal origin. *Plasmid* 28:130-145.
